# Interaction between Phrasal Structure and Vowel Tenseness in German: An Acoustic and Articulatory Study

**DOI:** 10.1177/00238309211064857

**Published:** 2022-01-13

**Authors:** Malte Belz, Oksana Rasskazova, Jelena Krivokapić, Christine Mooshammer

**Affiliations:** Humboldt-Universität zu Berlin, Germany; University of Michigan, USA; Haskins Laboratories, USA; Humboldt-Universität zu Berlin, Germany; Haskins Laboratories, USA

**Keywords:** Final lengthening, vowel tenseness, prosody, speech production, German, kinematics

## Abstract

Phrase-final lengthening affects the segments preceding a prosodic boundary. This prosodic variation is generally assumed to be independent of the phonemic identity. We refer to this as the ‘uniform lengthening hypothesis’ (ULH). However, in German, lax vowels do not undergo lengthening for word stress or shortening for increased speech rate, indicating that temporal properties might interact with phonemic identity. We test the ULH by comparing the effect of the boundary on acoustic and kinematic measures for tense and lax vowels and several coda consonants. We further examine if the boundary effect decreases with distance from the boundary. Ten native speakers of German were recorded by means of electromagnetic articulography (EMA) while reading sentences that contained six minimal pairs varying in vowel tenseness and boundary type. In line with the ULH, the results show that the acoustic durations of lax vowels are lengthened phrase-finally, similarly to tense vowels. We find that acoustic lengthening is stronger the closer the segments are to the boundary. Articulatory parameters of the closing movements toward the post-vocalic consonants are affected by both phrasal position and identity of the preceding vowel. The results are discussed with regard to the interaction between prosodic structure and vowel tenseness.

## 1 Introduction

Phrase-level prosody has been conceived of as an organizational structure above the level of the word that marks prominence and groups words into prosodic phrases (see, for example., Beckman, 1996; Shattuck-Hufnagel & Turk, 1996; Wagner & Watson, 2010). Prosodic phrases are hierarchically organized, with hierarchically higher phrases dominating lower phrases (Beckman & Pierrehumbert, 1986; Krivokapić, 2014; Shattuck-Hufnagel & Turk, 1996). There are various approaches for describing the prosodic hierarchy (for reviews, see Frota, 2012; Grice et al., 2005; Shattuck-Hufnagel & Turk, 1996). For German, the most widely used model is Grice et al. (2005), where two layers of phrasing in the prosodic hierarchy are assumed, namely the intermediate (ip) and the intonation phrase (IP). Phonetically, in addition to tonal properties, boundaries are marked predominantly by lengthening of boundary-adjacent acoustic segments and articulatory gestures (also referred to as final and initial lengthening), and often by pauses (Fletcher, 2010; Krivokapić, 2007; Wagner & Watson, 2010). Articulatory studies also found that there is reduced gestural overlap (Bombien et al., 2013; Byrd et al., 2000; Byrd & Saltzman, 1998) and an increase in magnitude of the gestures at boundaries (see, for example, Fougeron & Keating, 1997). The most consistent of these properties is final lengthening (cf. studies in the following paragraph). However, while final lengthening has been established in numerous languages (for reviews, see Fletcher, 2010 and Katsika, 2016), it is less clear whether all segments are affected by the boundary in a similar way. In this study, we will investigate the interaction between the phonemic and the phrasal layer with regard to vowel tenseness and phrasal structure for German. Furthermore, our study contributes to the broader understanding of acoustic and articulatory boundary properties in German. In this vein, we examine the extent of final lengthening (for which there are almost no studies for German) and we provide an exploratory, empirical study of the kinematic properties of the closing movement toward the phrase-final consonant. Specifically, the effect of boundaries on gestural magnitude is poorly understood, and, for German, there is only very limited data, namely only one study examining two speakers of Viennese German (Mücke & Hermes, 2007).

### 1.1 Temporal and spatial properties of prosodic boundaries

We start with a brief review of temporal and spatial properties of prosodic boundaries. Final lengthening at prosodic boundaries has been documented in many languages in articulatory and acoustic studies (e.g., for English, Byrd & Saltzman, 1998; Cho, 2006; Edwards et al., 1991; S. Kim et al., 2017; Lehiste, 1970, 1973; Turk & Shattuck-Hufnagel, 2000, 2007; Wightman et al., 1992; see Cho, 2015; Fletcher, 2010 and Vaissière, 1983 for an overview). Lengthening at prosodic boundaries increases with boundary strength, with hierarchically higher phrases having more lengthening than hierarchically lower phrases, mirroring its hierarchical organization (Byrd, 2000; Byrd & Saltzman, 1998; Cho, 2006; Fougeron & Keating, 1997; Tabain, 2003; Tabain & Perrier, 2005; Wightman et al., 1992). Another temporal property of IP boundaries is that they are often marked by pauses. For German few studies exist, but they consistently found final lengthening as an acoustic correlate of prosodic boundaries. For example, Peters (2003) investigated the acoustic properties of IP boundaries in German spontaneous speech and found final lengthening in 66% of turn-internal phrases and in 74% of turn-final phrases, mostly in combination with other phonetic parameters such as pauses or tonal breaks. In their study on syntax-prosody mapping Kentner and Fèry (2013) asked speakers to read sentences in which names were grouped into phrases indicated orthographically by brackets. Final lengthening and fundamental frequency (F0) were used as acoustic means for the disambiguation of the name groupings. Similarly, Petrone et al. (2017) conducted a production and perception study to investigate the role of three prosodic boundary cues for German: final lengthening, F0, and pauses. The findings of the production study regarding final lengthening showed that duration of the syllable-final vowel was longest at the phrase boundary.

The scope of lengthening is not restricted to the segment immediately preceding the boundary, but it extends to segments farther away, as a number of acoustic and articulatory studies have found (Berkovits, 1993a, 1993b, 1994; Byrd et al., 2006; Byrd & Riggs, 2008; Cambier-Langeveld, 1997; Cho et al., 2013; Katsika, 2016; J. Kim, 2020; Oller, 1973; Seo et al., 2019; Turk & Shattuck-Hufnagel, 2007; Turk & White, 1999). Furthermore, the lengthening of segments and gestures is not uniformly distributed, rather, the segments and gestures closest to the boundary lengthen more than segments farther away from it. This has been shown for various languages, for example, in Hebrew, Dutch, Greek, Japanese, and English (Berkovits, 1993a, 1993b; Byrd et al., 2006; Cambier-Langeveld, 1997; Katsika, 2016; Krivokapić, 2007; Seo et al., 2019; Shattuck-Hufnagel & Turk, 1998; see also Fougeron & Keating, 1997). There is evidence that the scope of lengthening is dependent on stress, such that final lengthening starts earlier when stress occurs earlier in the word compared to when it occurs later (Katsika, 2016; Katsika et al., 2014; Turk & Shattuck-Hufnagel, 2007; Turk & White, 1999; White, 2002; see also Berkovits, 1994; Byrd & Riggs, 2008; Oller, 1973; Seo et al., 2019).

For German, the scope of lengthening has not been systematically examined, although there is initial evidence that the same pattern holds. In a study of two speakers, Kohler (1983) found that words lengthen utterance finally, with the last syllable preceding a prosodic boundary lengthening the most, and lengthening decreasing farther away from the boundary. Mücke and Hermes (2007) investigated articulatory correlates of final lengthening for two speakers of Viennese German using articulography data (to our knowledge this is the only articulatory study to investigate lengthening in articulation for German). They found lengthening of consonantal closing movements closest to the boundary. While they did not directly examine this, their results also show some evidence of lengthening being stronger at the boundary than farther away from it.

The temporal properties of prosodic boundaries have been modeled with the π-gesture model (Byrd & Saltzman, 2003) that has been proposed within Articulatory Phonology (e.g., Browman & Goldstein, 1990, 1992; Goldstein et al., 2006). Within this model, the prosodic boundary is viewed as a prosodic gesture (π-gesture) which extends over an interval and is co-active with constriction gestures (e.g., vowel and consonant gestures) at the boundary. The π-gesture locally slows the utterance clock and in that way slows the rate of gestural activation of the co-active gestures. As a result, gestures at boundaries become temporally longer and less overlapped. The activation degree of the π-gesture determines the degree of clock-slowing and therefore the amount of lengthening of the co-active gestures. Hierarchically, higher boundaries have a stronger π-gesture activation and therefore gestures (and acoustic segments) show stronger boundary-related lengthening. The activation of the π-gesture also gradually increases as it approaches the end of a phrase and decreases farther away from it, leading to more lengthening closer to the boundary, and decreased lengthening farther away from it. Lengthening is also expected to affect all gestures (and segments) under the scope of the boundary.

We will use the π-gesture model to evaluate the scope of lengthening in our study since this model, as opposed to other prosodic approaches, can account for temporal properties of boundaries. Note that the predictions of the π-gesture model have been overwhelmingly supported for many languages (but see Turk & Shattuck-Hufnagel, 2007, and Rusaw, 2013, who find that the unstressed syllables between a stressed and the final syllable do not always lengthen). It should also be added that the π-gesture approach has recently been extended to account for (among others) some of the observed effects of lexical stress on the onset of final lengthening (Katsika et al., 2014). While the relationship between boundaries and prominence is not the focus of our study, we will return to this question in the discussion.

In addition to temporal properties, many studies have found some qualitative segmental modifications in direction of articulatory strengthening, both phrase-initially (Cho & Keating, 2001; Fougeron & Keating, 1997; Keating et al., 2004) and phrase-finally (Byrd et al., 2006; Fougeron & Keating, 1997; Keating et al., 1999; Tabain, 2003; Tabain & Perrier, 2005). For example, Fougeron and Keating (1997) found greater linguopalatal contact for consonants in phrase-initial position than in phrase-medial position, and, similarly, for vowels they find more contact phrase-medially than phrase-finally, indicating, in both cases, greater displacement. The phrase-final effects were less consistent than the phrase-initial effects. Tabain (2003) showed that the peak displacement of the vowel /a/ was affected by the prosodic hierarchy, that is before stronger prosodic boundaries the tongue body and jaw position were lower than before weaker prosodic boundaries. A systematic effect of boundary on displacement was found for Korean (J. J. Kim et al., 2019), such that, for up to two syllables (closing and opening movements of three consonants), there was more displacement of consonant movement phrase-finally compared to phrase-medially. Byrd et al. (2006) find inconsistent spatial effects, which varied across speakers. Overall then, there is evidence of an increase of phrase-final articulatory displacement, but it is less systematic than phrase-initially and less systematic than the boundary effects in the temporal domain. Working within the framework of Articulatory Phonology (e.g., Browman & Goldstein, 1989), Beckman et al. (1992) tested several predictions for jaw movements in English that might model kinematic changes for prosodic boundaries and pitch-accented words. The relationship between the duration, the amplitude and the peak velocity of a gesture can be described by the mass-spring model, that is, a spring with unit mass attached (see Browman & Goldstein, 1992; Saltzman & Munhall, 1989). Within this model, lengthening without changes of the movement amplitude are generated by a spring with a lower stiffness, which is further indicated by proportionally lower peak velocities. Beckman et al. (1992) found longer gestural durations and lower peak velocities without changes in displacement in phrase-final position which can be modeled as a spring with a lower stiffness in phrase-final position. Longer durations can also be modeled by a later phasing causing a smaller degree of gestural overlap, that is, a gesture can reach its gestural target without truncation. Beckman et al. (1992) assume this mechanism to underlie accentual lengthening. However, several studies found larger displacements and, in some cases, also larger velocities in pre-boundary position (cf. Byrd, 2000; Edwards et al., 1991; J. J. Kim et al., 2019). As described above, lengthening within the π-gesture model is generated by longer gestural activation intervals where the extent of lengthening decreases with the distance from the maximum of the π-gesture (Byrd & Saltzman, 2003). Therefore, whereas a lower gestural stiffness only predicts the local changes in kinematic parameters of the gesture adjacent to the boundary, the π-gesture approach can also account for the changes farther away from the boundary. Similar to the stiffness account, prolonging the activation interval lengthens the gestural duration, reduces the peak velocity and should not affect the displacement of the movement. As was shown in several simulations by Byrd and Saltzman (2003), the π-gesture approach does not affect the spatial properties directly, but only as a consequence of the temporal changes because the π-gesture also reduces the amount of intergestural overlap. As was pointed out by J. J. Kim et al. (2019), increasing the movement amplitudes can also result in larger peak velocities. Therefore, in both the stiffness account and the π-gesture model, changes in duration and peak velocity are predicted before boundaries, but only the π-gesture model can capture articulatory behavior, such as spatial expansion, less coarticulation and effects farther away from the boundary, by a single mechanism in a uniform model (for further comparison between models, see Byrd & Krivokapić, 2021; Byrd & Saltzman, 2003).

In their articulography study, Mücke and Hermes (2007) found for the two speakers of Viennese German that the displacement of the closing movement of the consonant of stressed CV syllables was larger phrase-finally, but only in disyllabic words and not in monosyllabic words. This is surprising since the stressed syllable was farther away from the boundary in the disyllabic than in the monosyllabic condition. Thus, although there are indications that prosodic structure may cause spatial changes, the evidence is less consistent and weaker than the temporal domain-final effects, and also less consistent than phrase-initial spatial effects.

### 1.2 The tense-lax contrast and stretchability

Even though this is not explicitly stated, most prosodic models do not take into account that there might be an interaction between phonemic identity, prosodic prominence, and edge effects, that is, the phonetic encoding of prosodic effects is not modeled as segment-sensitive (on this point see also Cho, 2011; Keating & Shattuck-Hufnagel, 2002). We will refer to this assumption as the “uniform lengthening hypothesis” (short ULH; note that it is also a prediction of the π-gesture model of Byrd & Saltzman, 2003 that the effect of the boundary is not gesture and segment specific). However, there is evidence for an interaction between phonemic identity and prosodic structure. For example, Cooper and Danly (1981) found that, for eight speakers of American English, lax /ɪ/ does not lengthen significantly in utterance-final position as opposed to tense /i/, which does, and further studies have demonstrated that other segments might be affected differently by prosodic boundaries as well (Cooper & Danly, 1981; Fougeron, 2001; Nakai et al., 2012; Tabain, 2003; for a review see Cho, 2011). Cluster-specific changes in overlap in phrase-initial position were found by Bombien et al. (2010) and Bombien et al. (2013).

For prominence, Hoole and Mooshammer (2002) and Mooshammer and Fuchs (2002) showed, for German, that lax vowels stretched only minimally for word stress or slower speech rates. Tense vowels, however, stretched in stressed syllables and compressed for fast speech rate (Hoole & Mooshammer, 2002). Evidence for this asymmetry was also found by Weirich and Simpson (2015): Phrase-level prominence (accented vs. unaccented) affected the duration of tense vowels in German to a greater degree than the duration of lax vowels. In addition, segments vary as to their affinity to be affected by speaking style. For example, in English, lax vowels are affected by clear versus conversational speaking style, that is they are stretched in clear style, but less so than tense vowels (Smiljanic & Bradlow, 2008). These studies indicate that there might be a difference in terms of segments’ stretchability in general, and in segments’ interaction with prosodic structure in particular.

In German, as in other Germanic languages, tenseness is correlated with quantity, that is tense vowels are about twice as long as lax vowels (see, for example, Hoole & Mooshammer, 2002; Jørgensen, 1969). The co-variation between vowel quality and quantity is restricted to the stressed position. In unstressed position, the durational differences between tense and lax vowels are negligible (Hoole & Mooshammer, 2002; Mooshammer & Fuchs, 2002) because tense vowels shorten in unstressed position whereas lax vowels maintain their duration. Apart from durational differences, most tense vowels are produced in a more peripheral position than lax vowels, as shown in acoustic and articulatory studies (Hoole & Mooshammer, 2002; Jørgensen, 1969; Mooshammer & Geng, 2008), except for the low vowel /a/ that is only distinguished by quantity and not by quality in German (for acoustics and perception, see Heike, 1972, and Sendlmeier, 1981, respectively). Lax vowels in CVC sequences are produced with shorter opening and closing movement durations and with smaller movement amplitudes for low vowels and larger amplitudes for high and mid vowels compared to their tense counterparts (see Hoole & Mooshammer, 2002).

Furthermore, there exists a distributional asymmetry concerning syllable structure: In stressed position, lax vowels can only occur in closed syllables, whereas tense vowels are not restricted in this way (for an overview see Vennemann, 1991). This conglomerate of characteristics has led to the view that tenseness is not a feature of the vowel but of the syllable. For example, Trubetzkoy (1939) suggested that tenseness is based on the type of the contact between the vowel and the coda consonant. For lax vowels, there is a *close contact* between the vowel and the following consonant that cuts off the vowel. Tense vowels can unfold fully because they are produced with a *loose contact* with the following consonant that is not obligatory. Apart from the syllable structure asymmetries, this also explains why tense vowels can be stretched while lax vowels are relatively stable in their duration: The latter vowels cannot lengthen because they have to be cut off by the following consonant (Trubetzkoy, 1938) and in his words lack the phonological feature *Dehnungsfähigkeit* (Engl. “ability to stretch”). Kinematic evidence for this view has been found for CVC sequences with identical consonants by Kroos et al. (1997) and Hoole et al. (1994) with a tighter coupling between the opening movement and the closing movement for lax nuclei compared to tense. Sievers (1901) and Vennemann (1991) used the term *Silbenschnitt* (Engl. “syllable cut”) prosody, based on the shape of the intensity curve. For both smoothly and abruptly cut syllables, the intensity rise (or “crescendo” after Vennemann, 1991) takes place during the vowel. For the abruptly cut syllables, the intensity decline falls in the following consonant, whereas for a smoothly cut syllables it occurs during the vowel. Even though this approach to tenseness is not often discussed in mainstream phonology, it can account for several properties at the same time.

To our knowledge, there are only two studies on German (Kohler, 1983; Mücke & Hermes, 2007) that analyzed words with tense and lax vowels separately at prosodic boundaries. Based on two speakers, Kohler (1983) found less extensive lengthening for words with lax vowels, but lengthening nevertheless occurred, indicating that lax vowels are not completely insensitive to prosodic variation. Mücke and Hermes (2007) showed for two speakers of Viennese German that the closing movement duration in phrase-final position was lengthened in mono- and in disyllabic target words with the vowels /a**ː**/ and /a/. Lengthening effects were restricted to the closing movement following tense vowels whereas after lax vowels the closing movement was only lengthened by 3 ms on average. The authors state that “[l]engthening [of the closing gesture] is not an option for the phonologically short vowel, since it would involve a risk of confusion with its long counterpart” (Mücke & Hermes, 2007, p. 1000).

### 1.3 Goals of the study

The primary goal of this study is to examine the effect of the prosodic boundary on tense and lax vowels in German. Based on previous studies we hypothesize that, due to their diminished stretchability, lax vowels are lengthened to a lesser degree in phrase-final position than tense vowels. This hypothesis is contrary to the assumptions of the “uniform lengthening hypothesis” which states that all gestures and segments should be affected by the boundary in a similar manner, that is, not only should all gestures lengthen, but the relative amount of lengthening should be comparable. We test these opposing predictions for acoustic vowel duration and the articulatory duration of the closing movement toward the consonant following the target vowels. Furthermore, the π-gesture model of Byrd and Saltzman (2003) predicts that lengthening is strongest at the boundary and decreases continuously with distance from the boundary. We examine whether this effect is found for the boundary-adjacent acoustic segments in our target words.

**Figure 1.**
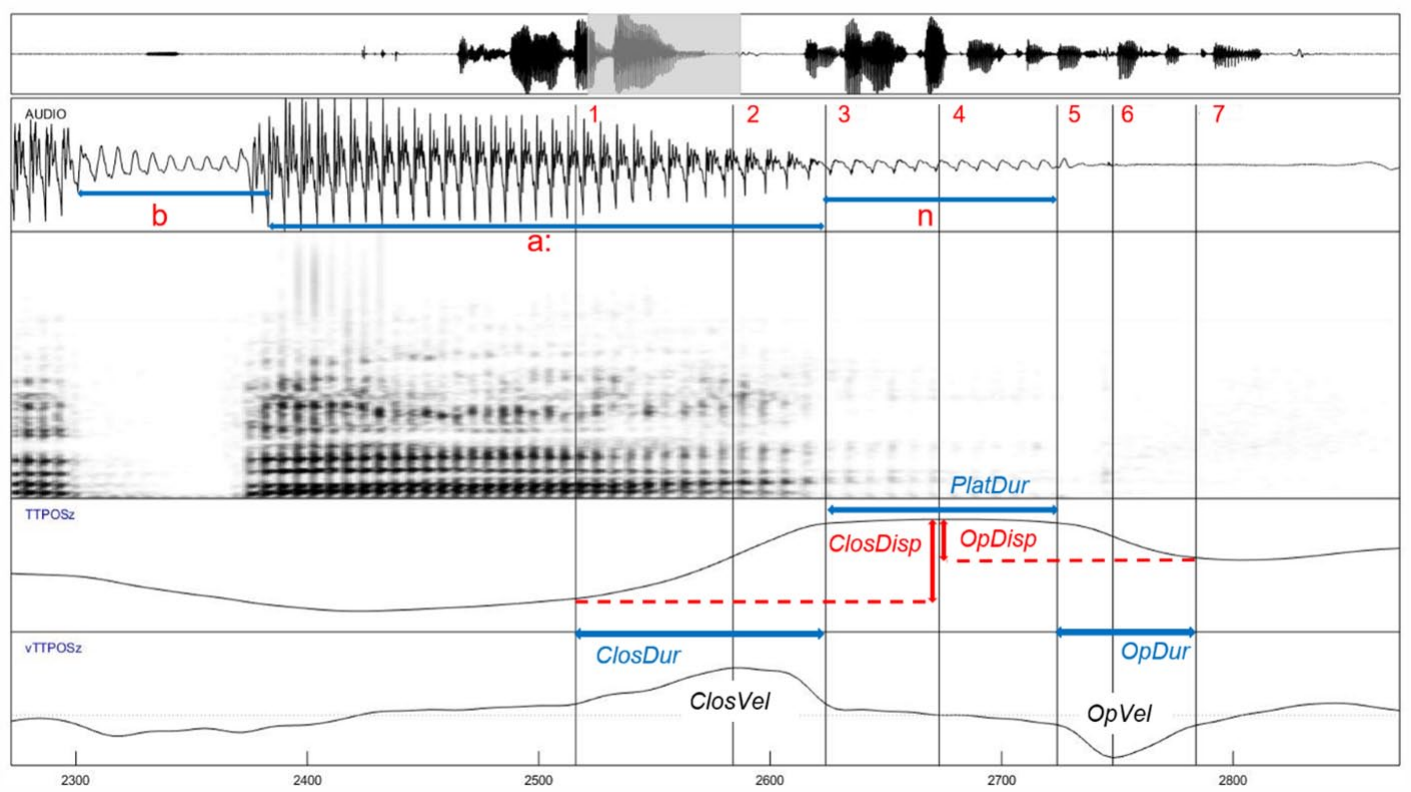
Labeling procedure for measuring kinematic parameters, exemplified for the articulatory movements for the pre-boundary consonant /n/ in the target word *Bahn*. The vertical tongue tip signal (TTPOSz) and its velocity (vTTPOSz) was labeled for (1) gesture onset, (2) peak velocity of closing movement, (3) plateau onset, (4) point of maximum constriction, (5) plateau offset, (6) peak velocity of opening movement, and (7) gesture offset. From this data the following parameters were derived: Closing and opening duration of the consonant movement (ClosDur, OpDur), plateau duration (PlatDur), and displacement of the closing and opening movement (ClosDisp, OpDisp).

These research questions will be addressed by investigating two sets of variables. First, in Section 3.1, acoustically measured segment durations will be analyzed for effects of tenseness and phrasal position. This will provide information on the range of the lengthening effect and the consistency across different phonemes. The second part in Section 3.2 will explore the effect of the boundary on kinematic properties of the tongue tip movement toward the consonant following the tense-lax vowel in the stressed syllable (for an explanation cf. Section 2.3.2), with the goal of contributing empirical knowledge of boundary-related processes. As has been shown above, many studies on final lengthening focus on analyzing articulatory parameters of the boundary-adjacent movements in order to gain a deeper and more fine-grained view into the underlying control mechanisms (e.g., Beckman et al., 1992; Byrd et al., 2000; Cho & McQueen, 2005; Edwards et al., 1991; Fougeron & Keating, 1997; Katsika, 2016; Mücke & Hermes, 2007; Tabain, 2003). Apart from this crucial insight, articulatory measurements also improve the accuracy of temporal measures. For example, the acoustically measured consonant duration does not always capture the duration of the constriction duration correctly prior to pauses (cf. the consonant movement in Figure 1 to the consonant in the spectrogram). For laterals and nasals, the consonantal release can also occur after voicing stopped.

## 2 Method

### 2.1 Participants and recording procedure

Ten German native speakers (5 male, 5 female) between 23 and 28 years without noticeable speech impairments participated in this study. Participants varied in their regional background with six participants from Berlin and the adjoining federal state of Brandenburg and four participants from Saxony, Bavaria, North Rhine-Westphalia, and Baden-Württemberg, respectively. Despite this variation, they did not show any salient traits of regional vernaculars. They were informed about the methods and recording procedure, but they were not aware of the goal of the study. After obtaining their consent, data about their age, gender, and language background were collected. None of the participants reported speech or hearing impairments. Participants received a payment of 10 EUR per half hour. The participants were seated in a sound-attenuated booth and instructed to read stimuli sentences from a computer screen. Stimuli were presented in blocks of five iterations in a randomized fashion, and the start of each stimulus was cued by a visual and an auditory signal. Participants were given the opportunity to rest between blocks if they wished. The experimenter was visible to the speakers throughout the experiment via a pane.

Acoustic data were recorded at 44.1 kHz using a shotgun microphone located in front of the speakers. Articulatory data were recorded simultaneously by means of electromagnetic articulography (EMA), using the articulograph AG 501 (Carstens Electronics, for details on accuracy cf. Savariaux et al., 2017). The sampling rate of the articulatory data was 1250 Hz which was downsampled to 250 Hz for post-processing in MATLAB (version R2013b). This method allowed us to record the tongue, jaw and lip movements over time in three-dimensional space by means of sensors attached to tongue tip (TT), tongue mid (TM), and tongue back (TB), the lower incisors (JAW), and the upper and lower lips. These sensors were glued to the articulators by medical adhesive. In addition, the attachment of the tongue sensors was fortified with dental cement. To compensate for participants’ head movements, four reference sensors were placed just above the upper incisors, on the nasion and on the left and right mastoid part of the temporal bone. In total, 10 sensors were used simultaneously during the experiment. In addition, three sensors were attached to a set square to record the bite plane just before the end of the experiment. After low-pass filtering the reference sensors at 20 Hz, the data were corrected for head movement and then rotated and translated to the recorded bite plane. Due to technical problems, the reference sensor data of two participants could not be translated to the bite plane, so that the data were rotated and translated to a plane between the upper incisors and the nasion instead. The EMA recordings have been approved by the ethics committee of the German Linguistic Society.

### 2.2 Material

The stimuli consisted of six minimal pairs of target words differing in vowel tenseness (cf. Table 1). Four of the minimal pairs were monosyllabic, and two were disyllabic. Note that since lax vowels are restricted to closed syllables, both tense and lax vowels had to occur in closed syllables in monosyllabic words. In disyllabic words, the tense-lax vowels occur in the non-final (penultimate) syllable, and we assume the intervocalic singleton consonants after stressed lax vowels (e.g., /t/ in *Mitte* /mɪṭə/ “mid”) to be ambisyllabic in German. The tense vowels in the corresponding disyllabic minimal pairs occur in open syllables. The target words were embedded in carrier sentences for two boundary conditions as shown for the target word *Bahn* in phrase-medial (1) and phrase-final position (2).

(1) Ich fuhr mit der Bahn am Donnerstag. Am Mittwoch wurde noch gestreikt.
“I took the train on Thursday. On Wednesday, there was still a strike.”
(2) Ich fuhr mit der Bahn. Am Donnerstag musste ich in Frankfurt sein.
“I took the train. On Thursday, I had to be in Frankfurt.”

**Table 1.** Mono- and disyllabic minimal pairs for tense and lax vowels.

	Monosyllabic				Disyllabic	
tense	Bahn [ba**ː**n] ‘train’	Beet [be**ː**t^h^] ‘bed (bot.)’	Ruhm [ʁu**ː**m] ‘fame’	Stiel [ʃti**ː**l] ‘stipe’	Miete [mi**ː**.t^h^ə] ‘rent’	Hüte [hy**ː**.t^h^ə] ‘hats’
lax	Bann [ban] ‘ban’	Bett [bɛt^h^] ‘bed’	Rum [ʁʊm] ‘rum’	still [ʃtɪl] ‘quiet’	Mitte [mɪṭ^h^ə] ‘middle’	Hütte [hʏṭ^h^ə] ‘hut’

The carrier sentences were identical in the material preceding the target word and for at least two words after the target word. We designed the sentences such that we expected, based on native speaker intuitions, that the target words will be pitch-accented. Modifying the position of the target word within the phrase could also affect the type of accent, that is, prenuclear or nuclear accent in phrase-medial position and nuclear in phrase-final position. Based on existing research it is not known whether this difference in pitch accent type affects the duration of the words. We come back to this in the discussion. The full list of stimuli is given in Table A1. The stimuli were mixed with filler sentences in order to distract participants from the prosodic pattern. In total, there were 24 stimuli and 9 filler sentences. All sentences were repeated five times. The number of presented stimuli thus comprised 8 monosyllabic and 4 disyllabic target words uttered in 2 phrasal conditions and with 5 repetitions by 10 participants, amounting to 800 monosyllabic and 400 disyllabic target words.

### 2.3 Measurements

#### 2.3.1 Acoustic measurements

The recordings were pre-aligned with the stimuli words using WebMAUS (Kisler et al., 2017). The transcription and its alignment was checked and corrected with Praat (Boersma & Weenink, 2019). Every segment of each stimulus word was annotated on an interval tier, marking stop closure and VOT, vowels, fricatives, and nasals, by cycle and at zero crossings of the acoustic signal. The end of F2 of the preceding vowel as well as abrupt changes in the spectrogram were used as annotation criteria. To discern /i/ and /ɪ/ from the following laterals, the end of the F3 transition (when applicable) and changes in the envelope are used. Another interval tier was added to annotate whether the participants realized the intended prosodic condition (see Section 2.4). All Praat TextGrids were converted into an EMU speech database (Winkelmann et al., 2017) and analyzed with the package *emuR* (Winkelmann et al., 2016) in *R* (R Core Team, 2018). We examined this question in the acoustic signal only as our stimuli were designed to elicit tense and lax vowels, hence they do not lend themselves to systematic kinematic labeling except for the closing movement from the primary stressed vowel to the following consonant (s. 2.3.2). For example, *Beet* [be**ː**t^h^] ‘bed (botanical)’ was segmented into onset [b], nucleus [e**ː**], and coda [t^h^] together with aspiration, when present. Disyllabic target words were segmented into onset and nucleus of the first syllable (O1 and N1), as well as into onset and nucleus of the second syllable (O2 and N2, respectively). Words containing a lax vowel in the first syllable were also labeled that way, although we assume intervocalic singleton consonants after stressed lax vowels to be ambisyllabic in German.

#### 2.3.2 Articulatory measurements

Articulatory data were analyzed using *mview* (Tiede, 2005), a MATLAB-based tool which allows for semi-automatic labeling of kinematic parameters of an articulatory gesture. We analyzed the movement toward the final consonant in the stressed syllable as a fairly good approximation for the properties of the vowel.^1^ The tongue tip signal was analyzed for the alveolar consonants /t, n, l/. For the bilabial consonant /m/, the lip aperture signal (LA) was used. LA was calculated as the Euclidean distance between upper and lower lip signals. To quantify the effect of final lengthening, opening and closing gestures of the last consonant in the target words were used, for example, /t/ in *Bett* ‘bed’ or *Hütte* ‘hut’. Note that the last consonant of the disyllabic words is farther away from the boundary than the last consonant of the monosyllabic word. Movements of the consonantal articulator were labeled for different timepoints and intervals using a 20% threshold criterion of the maximal tangential velocity. The closing movement duration (ClosDur) toward the final consonant and the opening movement duration (OpDur) were measured as the time span of closing movement from gesture onset to maximum constriction (plateau onset) and opening movement from plateau offset to the gesture offset, respectively (see Figure 1). Plateau duration (PlatDur) was defined as the timespan between plateau onset and plateau offset, corresponding to the constriction or closed phase. The peak velocities were measured for the tongue tip and the LA signals of the closing (ClosVel) and the opening (OpVel) movements. The kinematic variables of the opening movements were not included in the further analysis, but—as explained in Section 2.4—used as a criterion for data exclusion. Displacements of opening and closing movements were calculated as the Euclidean Distance from the point of maximum amplitude to the onset of the closing movement (ClosDisp) or to the offset of the opening movement (OpDisp).

### 2.4 Data processing

All utterances were annotated regarding production errors such as hesitations, slips of the tongue, and repetitions with repairs in the vicinity of the target words, and such stimuli were excluded. Furthermore, we verified whether the speakers produced the targeted prosodic structure. A research assistant, naive to the purposes of the study, was trained by the first author on the data of one of the subjects to audio-visually inspect and label all data for the presence of prosodic boundaries identified by a perceptible break and the presence of an edge tone. Our main criterion was a perceptible prosodic break, and thus our data might include some intermediate phrases, though we consider this unlikely, given that these were all clearly identifiable breaks. For further analyses, boundaries were taken to be phrase-medial or phrase-final based on how the speakers produced them (and not based on what the targeted boundary was). 16 utterances of one speaker had a different prosodic structure than the targeted structure and were re-categorized into the respective other condition. Four sentences with production errors were excluded. Table 2 shows the results of the verification process, excluding 2% of the data. The research assistant also checked that all test words carried a pitch accent.

**Table 2.** Results of the prosodic annotation for the phrase-medial and phrase-final positions for all 10 participants. Divergent realizations are defined as (b) no noticeable boundary realization in phrase-final position, (c) unexpected boundary realization in phrase-medial position, and (d) production errors. The calculation path for ∑ is given in parentheses.

		i. Phr.-medial	ii. Phr.-final	∑
a.	As expected	589	591	1180
b.	No noticeable boundary	–	6	6
c.	Unexpected boundaries	10	–	10
d.	Production errors	3	1	4
∑	Target words analyzed	582 (a.i−c.i−d.i+b.ii)	594 (a.ii−b.ii−d.ii+c.i)	1176

Utterances that contained extralinguistic noise had to be excluded from the acoustic, but not from the articulatory analysis. Labeling of the acoustic signal revealed that some segments had been elided by the participants. In total, we included 784 syllable onsets, nuclei, and codas in the monosyllabic analysis, as well as 392 onsets of the first and second syllable and 392 onsets of the first syllable in the disyllabic analysis. The latter included 391 nuclei of the second syllable, as Schwa has been elided in one case of *Miete*. The total amount of excluded acoustic data is 2.02%.

During articulatory labeling it was observed that for the phrase-final condition the opening movement was highly variable, especially in the case of nasals in the target words *Bahn* (‘train’) and *Bann* (‘ban’). In these cases, speakers did not release the articulatory gesture for the preboundary consonant /n/ and instead maintained the tongue tip at the alveolar ridge until the postboundary gesture. The consequence of an unreleased consonantal constriction is no detectable opening movement, so the offset of the plateau cannot be measured consistently. In order to obtain a consistent measure for the plateau duration, cases with a preboundary consonant opening displacement of less than 1 mm were excluded. This threshold is also applied to closing displacement, as this amplitude is close to the accuracy of the system, affecting, in sum, 36 mono- and 8 disyllabic stimuli. In total, 748 monosyllabic and 385 disyllabic stimuli are included in the articulatory analysis. The total amount of excluded articulatory data is 5.58%.

### 2.5 Statistics

For the acoustic analysis, we took the duration of the respective syllable constituent (onset, nucleus, coda) as the dependent variable. For the articulatory analysis, the dependent variables were articulatory closing duration, plateau duration, closing displacement, and closing velocity. For both domains, fixed effects were tenseness (lax vs. tense), phrasal position (phrase-medial vs. phrase-final), and their interactions. Participants and target words were included as random effects, with by-participant random slopes for the predictors, where possible.

Linear mixed-effects (LME) models for the respective dependent variables were calculated in *R* (R Core Team, 2018) using the *lme4* package v.1.1-12 (Bates et al., 2015). Fixed factors are treatment coded. For all LME models the unnormalized data was used, as speech rate is already accounted for in the model structure via the by-participant random intercepts and by-participant random slopes for the predictors. Speech rate ranged from 4.8 syllables per second (slowest participant) to 6.3 syllables per second (fastest participant), as calculated with the *sylly* package (Michalke, 2017).

Maximal models were built, taking all predictors into account. Interactions are only included if the model converges and if they significantly improve the model. This is evaluated comparing AIC (Akaike, 1974) and using likelihood-ratio tests. By-participant and by-word random intercepts as well as by-participant and by-word random slopes for the predictors are included, thus taking the repetition of the target words into account. Random slopes are stepwise reduced if the model does not converge. In some cases, the random slopes had to be removed for the model to converge. Outliers were excluded based on the visual inspection of quantile-quantile plots of the fitted values and the residuals for each model, in order to achieve normally distributed residuals (Baayen, 2008). The number of included stimuli is given in the model reports. Further model criticism included the visual inspection for heteroskedasticity of the residuals. Additional p-values are calculated with the package *lmerTest* v.3.0-1 (Kuznetsova et al., 2017). Marginal ( $R_{m}^{2}$ ) and conditional coefficients of determination ( $R_{c}^{2}$ ) were calculated for each model, using the package *MuMIn* v.1.42.1 (Barton, 2018). Marginal $R_{m}^{2}$ is a measure for the variance explained by the fixed factors only, conditional $R_{c}^{2}$ gives the variance explained by both fixed and random factors (Nakagawa & Schielzeth, 2013). For the interpretation of the model outcome multiple post-hoc comparisons with Tukey-adjusted p-values were calculated to validate whether an effect applied to all factor levels, using the package *emmeans* v.1.4 (Lenth, 2019). These tests are included in the data supplement. All models may be replicated with help of the supplementary data in Belz et al. (2021).

## 3 Results

As explained in the introduction, we examine whether phrasal position affects lax vowels less than tense vowels. In the acoustic study we further evaluate whether lengthening is stronger for segments closer to the boundary, following Byrd and Saltzman (2003). First, the acoustic results for the mono- and disyllabic stimuli will be reported, followed by the articulatory results for the kinematic parameters.

### 3.1 Acoustic

#### 3.1.1 Monosyllabic target words

**Figure 2.**
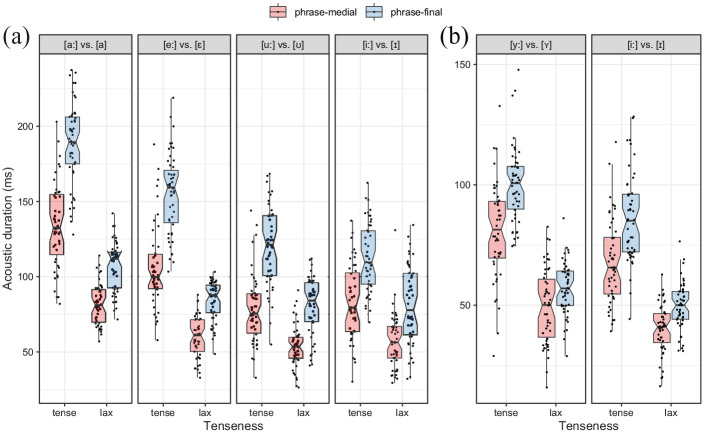
Acoustic duration of (a) vowels in monosyllabic and (b) stressed vowels in the first syllable of disyllabic words over tenseness and condition.

**Figure 3.**
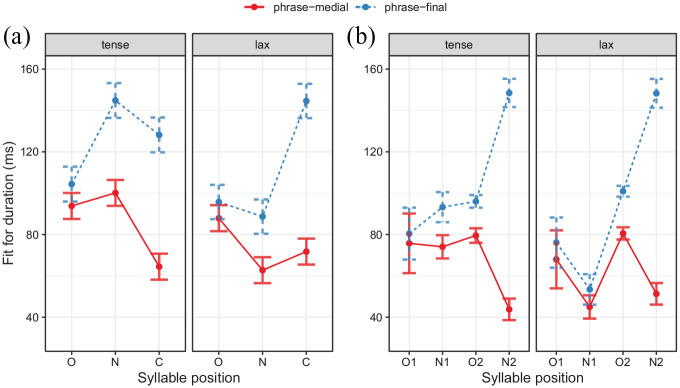
Fitted acoustic duration. (a) Monosyllabic items with syllable onsets (O) /b ʁ ʃt/, nuclei (N) /a**ː**-a e**ː**-ɛ u**ː**-ʊ i**ː**-ɪ/, and codas (C) /n t m l/. (b) Disyllabic items with first onsets (O1) /h m/, first nuclei (N1) /y**ː**-ʏ i**ː**-ɪ/, second onset (O2) /t/, and second nucleus (N2) /ə/.

Figure 2(a) displays the results of the acoustic duration of tense and lax vowels (e.g., /a**ː** a/ in *Bahn/Bann*). Table 3 contains the outcome of a model of syllable constituents, whose effects are visualized in Figure 3(a). Nuclei (containing the vowels) undergo significant lengthening in the lax and tense condition (all significant post hoc comparisons are given in Table A2 in the Appendix). In general, lax vowels lengthen by 26 ms (cf. row 1), and tense vowels by 45 ms (cf. row 15). In absolute terms, lax vowels in *Bann, Bett, Rum, still* are lengthened less than tense vowels in *Bahn, Beet, Ruhm, Stiel* (cf. Figure 2[a]). In relative terms, tense vowels lengthen slightly more, 45% than lax vowels, 41%. More proportional lengthening was found for three of the four word pairs, with the exception of the word pair for /i**ː** - ɪ/ that shows less proportional lengthening for tense /i**ː**/, 38%, than for lax /ɪ/, 43%. An additional model was calculated to test for the proportional amount of vowel lengthening. The model outcome in Table 4 shows that there is no significant difference between the proportional lengthening of tense and lax vowels.

**Table 3.** Model predictions for the acoustic duration of syllable position in monosyllabic words (standards error in parentheses) for the fixed effects phrasal position (phrase-medial vs. phrase-final), syllable constituent (*sylcon*, O = onset, *N* = nucleus, C = coda), and tenseness (tense vs. lax).

	Duration in monosyllabic words
(Intercept)	93.8 (6.3)***
positionphrase-final	10.6 (4.7)*
sylconN	6.3 (3.3)
sylconC	−29.4 (3.3)***
tensenesslax	−5.9 (8.1)
positionphrase-final: sylconN	34.1 (4.6)***
positionphrase-final: sylconC	53.2 (4.6)***
positionphrase-final: tensenesslax	−2.7 (5.4)
sylconN: tensenesslax	−31.5 (4.6)***
sylconC: tensenesslax	13.2 (4.6)**
positionphrase-final: sylconN:tensenesslax	−16.1 (6.5)*
positionphrase-final: sylconC:tensenesslax	11.9 (6.5)
AIC	22939.7
Num. obs.	2342
Num. groups: subject	10
Num. groups: word	8
$R_{m}^{2}/R_{c}^{2}$	0.29/0.41

AIC: Akaike information criterion.

*** *p* < .001, **p < .01, *p < .05.

**Table 4.** Model predictions for the proportional lengthening of vowels in mono- and disyllabic words (standards error in parentheses) for the fixed effects tenseness (tense vs. lax) and random intercepts for subjects and word pairs.

	Monosyllabic %	Disyllabic %
(Intercept)	48.3 (6.0)***	29.8 (5.1)***
tensenesslax	−5.8 (4.8)	−6.6 (7.2)
AIC	732.5	361.6
Num. obs.	80	40
Num. groups: subject	10	10
$R_{m}^{2}/R_{c}^{2}$	0.01/0.3	0.02/0.02
Num. groups: wordpair	4	2

AIC: Akaike information criterion.

*** *p* < .001, **p < .01, *p < .05.

**Figure 4.**
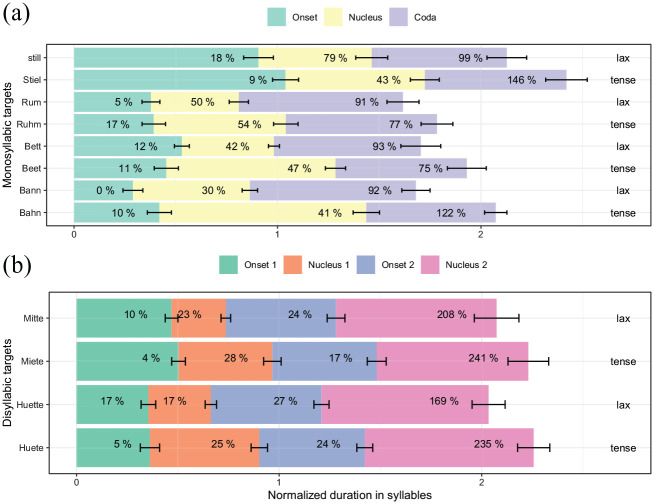
Acoustic mean duration of syllable constituents in phrase-final conditions (error bars show standard deviations), with numbers indicating the mean proportional lengthening in percent from medial to final. (a) Monosyllabic items with onsets /b ʁ ʃt/, nuclei /a**ː**-a e**ː**-ɛ u**ː**-ʊ i**ː**-ɪ/, and codas /n t m l/. (b) Disyllabic items with first onsets /h m/, first nuclei /y**ː**-ʏ i**ː**-ɪ/, second onsets /t/, and second nucleus /ə/.

Next, we evaluate whether segments closer to the boundary lengthen to a higher degree than segments farther away. In absolute terms, the effect of the boundary is strongest at the coda, smaller for nuclei and not significant for onsets, as can be seen from Figure 3(a). Figure 4(a) shows the mean onset, nucleus, and coda duration of the target words in phrase-final position. The lengthening proportion is calculated for each syllabic constituent over all participants and repetitions and always refers to the mean duration of its respective phrase-medial constituent (which is itself not shown in Figure 4). For example, the mean duration for /a**ː**/ (yellow) in tense *Bahn* is lengthened by 41% phrase-finally in comparison to its mean phrase-medial duration. The word pair *Stiel/still* is the only pair with two consonants in the onset and therefore shows longer absolute durations than the other onsets; the proportional values for lengthening of /ʃt/, however, behave similarly to singleton onsets. More lengthening can be observed for the nucleus, with 45% on average for tense and 41% for lax vowels. Codas show an even higher lengthening proportion than nuclei, stretching between 75% and 151%. No clear pattern can be observed for the proportional lengthening of codas as a function of tenseness. Thus, lengthening reaches up to the word onset, but segments closer to the boundary are in general lengthened to a higher degree than segments farther away from the boundary.

In line with the expectations, lax vowels do lengthen significantly less than tense vowels in final position, at least in mean absolute terms and without a closer inspection of the distinction of /i**ː**ɪ/ . This is not surprising, as phrase-medial tense vowels are already longer than phrase-medial lax vowels (s. Table 10, Row 4). Proportionally, however, lax vowels do stretch in a similar way than tense vowels, which is contrary to the hypothesis of diminished stretchability. Finally, referring to Figure 3(a), the phonological length contrast is not threatened by the lengthening of lax vowels. Although their duration in phrase-final positions is similar to the duration of phrase-medial tense vowels, their usage cannot be assumed to be interchangeable, as lengthening depends on the context of a phrase boundary.

#### 3.1.2 Disyllabic target words

To test the ULH for disyllabic target words, we compare the acoustic duration of tense and lax vowels (e.g., /i**ː**ɪ/ in *Miete/Mitte*), see Figure 2(b). Compared to the monosyllabic words, the picture is less clear, as the lax vowel [ɪ] in *Mitte* undergoes lengthening, but [ʏ] in *Hütte* does not. Table 5 contains the outcome of a model over syllable constituents, whose effects are visualized in Figure 3(b) (all significant post hoc comparisons are given in Table A3 in the Appendix). Lax vowels (N1) are not significantly affected by lengthening (8 ms). Tense vowels, however, lengthen significantly by 19 ms (cf. Table 11, Row 1). This effect is smaller and less consistent that for the vowels in monosyllabic words which can be attributed to the farther distance from the boundary.

**Table 5.** Model predictions for the acoustic duration of syllable position in disyllabic words (standard errors in parentheses) for the fixed effects phrasal position (phrase-medial vs. phrase-final), syllable position (*sylcon*, O1/O2 = first/second onset, N1/N2 = first/second nucleus), and tenseness (tense vs. lax).

	Duration in disyllabic words
(Intercept)	75.7 (13.8)***
positionphrase-final	4.7 (2.9)
sylconN1	−1.7 (18.1)
sylconO2	3.8 (12.0)
sylconN2	−32.0 (17.6)
tensenesslax	−7.8 (19.0)
positionphrase-final: sylconN1	14.5 (3.1)***
positionphrase-final: sylconO2	11.8 (3.1)***
positionphrase-final: sylconN2	100.0 (3.1)***
positionphrase-final: tensenesslax	3.4 (4.0)
sylconN1: tensenesslax	−21.3 (25.6)
sylconO2: tensenesslax	8.8 (16.9)
sylconN2: tensenesslax	15.3 (24.9)
positionphrase-final: sylconN1:tensenesslax	−14.1 (4.4)**
positionphrase-final: sylconO2:tensenesslax	0.4 (4.4)
positionphrase-final: sylconN2:tensenesslax	−11.1 (4.5)*
AIC	12857.9
Num. obs.	1541
Num. groups: subject	10
Num. groups: word	4
$R_{m}^{2}/R_{c}^{2}$	0.68/0.81

AIC: Akaike information criterion.

*** *p* < .001, **p < .01, *p < .05.

As for monosyllabic words, segments closer to the boundary exhibit a higher amount of lengthening than segments farther away (s. Figures 3[b] and 4[b]). No significant effects are found for the absolute durations of the word-initial onsets (O1). In relative terms they lengthen only slightly, between 5% and 17%. As mentioned above, only penultimate tense vowels (N1) show a significant lengthening in absolute terms (19 ms). Proportionally, tense vowels stretch between 25% (/y**ː**/) and 28% (/i**ː**/), lax vowels between 17% (/ʏ/) and 23% (/ɪ/), which is not significant as shown in Table 4. Word-medial consonants (referred to as O2) lengthen significantly between 17 and 20 ms (cf. Table 11, Rows 6 and 22). Word-medial consonants show a similar lengthening proportion as the preceding vowels. Schwa vowels (N2) lengthen significantly between 97 ms and 105 ms (cf. Table 11, Rows 13 and 24). Proportionally, they lengthen between 169% in lax *Hütte* and 241% in tense *Miete*. Although their mean absolute duration of 149 ms ( s = 38.3) is similar to the mean coda duration in monosyllabic words (136 ms, s = 31), their extensive amount of proportional lengthening can be explained by the short absolute duration of schwa vowels in the phrase-medial position.

Thus, although lengthening reaches up to the penultimate nucleus in disyllabic words, only tense vowels are significantly lengthened, and only so in absolute terms. Penultimate lax vowels are not lengthened significantly neither in absolute nor proportional terms. In this respect, the hypothesis that lax vowels are stretched to a lesser degree than tense vowels can be confirmed for the penultimate stressed position in disyllabic words. As for monosyllabic words, the phonological tense-lax contrast is not at risk.

### 3.2 Articulatory results

In the following section, we investigate the underlying production mechanisms by analyzing the kinematic parameters of the closing consonant movement, as the extent of lengthening cannot be measured in the acoustic signal following the offset of voicing (cf. Figure 1). In Sections 3.2.1 and 3.2.2, we present the results for all four dependent variables (closing duration, plateau duration, closing displacement and closing velocity) for the final consonant.

#### 3.2.1 Monosyllabic target words

**Figure 5.**
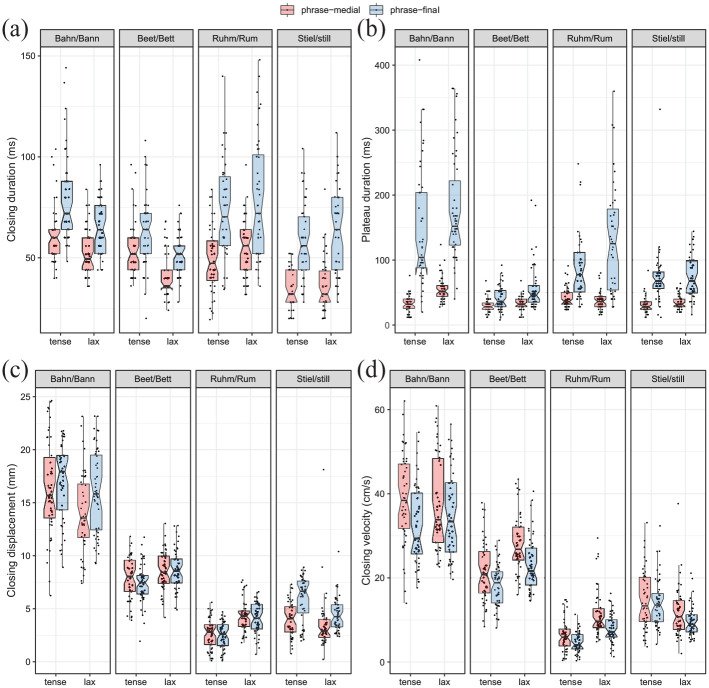
Articulatory variables for the last consonant segment in each monosyllabic word pair: (a) closing duration, (b) plateau duration, (c) closing displacement, and (d) closing velocity.

The data for the kinematic parameters of the articulatory movement toward the final consonant for each of the dependent variables (closing duration, plateau duration, closing displacement, and closing velocity) are visualized in Figure 5. The model effects with predictors phrasal position and tenseness are shown in Figure 7 and model predictions are given in Table 6. As shown none of the measurements are affected by tenseness, which means that lengthening of the closing movement affects the closing and plateau duration in roughly the same way after both tense and lax vowels, which can be observed in Figure 5(a) and (b). Closing and plateau duration are significantly longer in phrase-final than in phrase-medial position (cf. Figure 7[a] and [b]).

**Table 6.** LME model predictions for the articulatory measurements of closing movement duration (ClosDur), plateau duration (PlatDur), closing movement displacement (ClosDisp), and closing movement velocity (ClosVel) of the last consonant in all monosyllabic word pairs (standard error in parentheses) for the fixed effects phrasal position (phrase-medial vs. phrase-final) and tenseness (tense vs. lax).

	ClosDur (log)	PlatDur (log)	ClosDisp	ClosVel
(Intercept)	3.8 (0.1)***	3.4 (0.2)***	7.6 (2.9)*	20.5 (6.6)*
positionphrase-final	0.3 (0.0)***	0.8 (0.0)***	0.6 (0.2)*	−3.2 (0.9)**
tensenesslax	−0.1 (0.1)	0.2 (0.2)	−0.1 (4.0)	1.7 (9.0)
AIC	263.1	1052.9	3542.9	4936.5
Num. obs.	760	748	783	782
Num. groups: subject	10	10	10	10
Num. groups: word	8	8	8	8
$R_{m}^{2}/R_{c}^{2}$	0.19/0.49	0.34/0.58	0/0.88	0.02/0.87

AIC: Akaike information criterion.

*** *p* < .001, **p < .01, *p < .05.

Next, changes for the kinematic parameters displacement and peak velocity of the closing movement are considered. In the current study, the overall model shows that the closing displacement is significantly larger in phrase-final than in phrase-medial position, whereas the closing velocity is significantly slower in phrase-final than in phrase-medial position (effects are visualized in Figure 7[c] and [d]). This is contrary to the predictions by Beckman et al. (1992) as explained in Section 1.1. However, the overall model as calculated in Table 6 neglects the fact that tense-lax pairs differ in displacement and velocity in a non-uniform way. Because lax vowels are more centralized than tense vowels the displacement differences depend on vowel height and consonantal context (cf. Figure 5[c] and [d]).

Follow-up models are calculated for closing displacement and velocity to assess the differing vowel heights that co-vary with word pairs. Model predictions are given in Table 7. Although the overall model predicts a larger displacement of the consonants in phrase-final position (viz. Figure 7[c]), this effect is only significant for the vowel pair /i**ː**—ɪ/ in *Stiel/still*.

**Table 7.** LME model predictions for the articulatory measurements of closing movement displacement (ClosDisp) and closing movement velocity (ClosVel) of the last consonant in all monosyllabic word pairs (standard error in parentheses) for phrasal position (phrase-medial vs. phrase-final) and tenseness (tense vs. lax) in all monosyllabic target words.

	ClosDisp (mm)			
	Bahn/Bann	Beet/Bett	Ruhm/Rum	Stiel/still
(Intercept)	16.1 (1.3)***	7.9 (0.5)***	2.7 (0.4)***	4.2 (0.4)***
positionphrase-final	1.2 (0.7)	−0.3 (0.3)	−0.2 (0.1)	1.6 (0.3)***
tensenesslax	−1.6 (0.2)***	0.8 (0.2)***	1.6 (0.1)***	−1.3 (0.2)***
AIC	853.2	728.1	575.5	710.8
Num. obs.	198	193	193	195
Num. groups: participant	10	10	10	10
$R_{m}^{2}/R_{c}^{2}$	0.06/0.83	0.05/0.46	0.25/0.66	0.29/0.53
	ClosVel (cm/s)			
(Intercept)	38.1 (3.0)***	22.0 (1.7)***	6.4 (0.8)***	14.3 (1.1)***
positionphrase-final	−4.7 (1.6)**	−4.0 (0.6)***	−2.1 (0.3)***	−1.0 (0.6)
tensenesslax	0.4 (0.8)	5.6 (0.6)***	3.6 (0.3)***	−3.5 (0.6)***
AIC	1290.1	1124.5	850.1	1091.8
Num. obs.	198	193	187	191
Num. groups: participant	10	10	10	10
$R_{m}^{2}/R_{c}^{2}$	0.05/0.75	0.22/0.7	0.28/0.64	0.12/0.46

AIC: Akaike information criterion.;

*** *p* < .001, **p < .01, *p < .05.

For tenseness the minimal pairs show distinct behaviors. Contrary to tenseness showing no significant effect on displacement of the consonant movement in the overall model (viz. Table 6), there are significant differences when the word pairs are considered separately. On the one hand, displacements for lax /a/ and /ɪ/ are significantly smaller than tense /a**ː**/ and /i**ː**/. On the other hand, displacements for lax /ɛ/ and /ʊ/ are significantly larger than tense /e**ː**/ and /u**ː**/. Closing velocity slows down in final position, except for consonant /l/ following /i**ː**—ɪ/. Considering tenseness, closing velocity speeds up significantly for lax /ε/ and /ʊ/, which can be attributed to the larger Euclidean distance between the vowel target and the target of the final consonant, compared to their tense counterparts /e/ and /u/. For the gesture preceding /l/ displacement is reduced after the lax vowel. In accordance, the peak velocity is also reduced after lax /ɪ/ compared to tense /i**ː**/.

#### 3.2.2 Disyllabic target words

**Figure 6.**
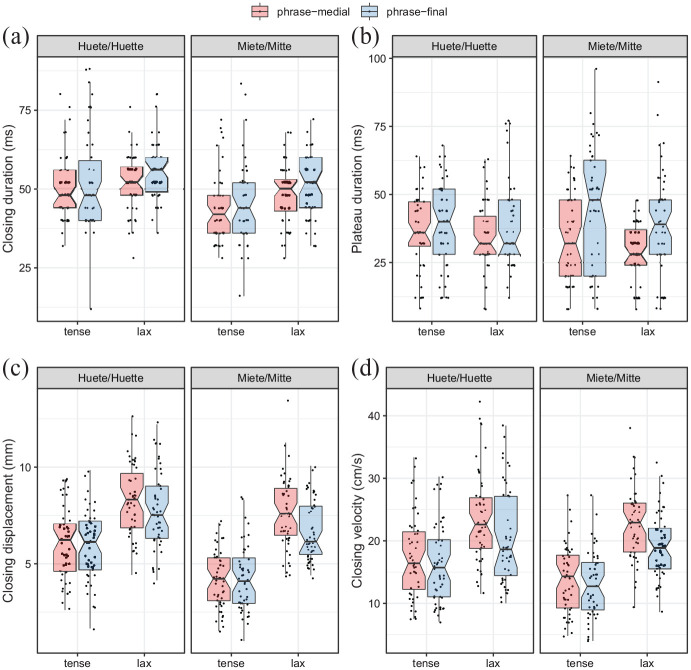
Articulatory variables closing duration, plateau duration, closing displacement and closing velocity for [t] in each disyllabic word pair.

**Figure 7.**
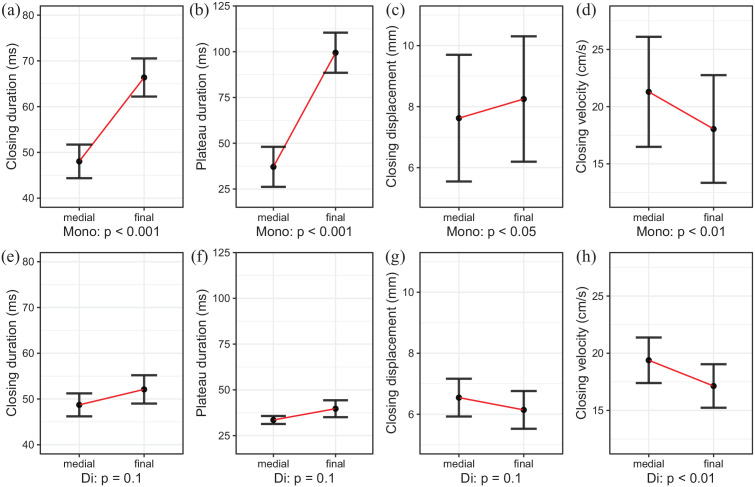
Effects of closing duration, plateau duration, closing displacement, and closing velocity of alveolar consonants in monosyllabic words (a-d) and disyllabic target words (e-h).

The data for each of the dependent variables closing duration, plateau duration, closing displacement and closing velocity for the articulatory movements toward the medial consonant /t/ are visualized in Figure 6, and the model effects are given in Figure 7 and Table 8. Note that compared to the monosyllabic words, the closing movement of /t/ in the case of the disyllabic words is by one syllable farther away from the boundary, therefore we expect a smaller boundary effect here, as was the case for the acoustic results.

**Table 8.** LME model predictions for the articulatory measurements of closing movement duration (ClosDur), plateau duration (PlatDur), closing movement displacement (ClosDisp), and closing movement velocity (ClosVel) of the last consonant in all disyllabic word pairs (standard error in parentheses) for the fixed effects phrasal position (phrase-medial vs. phrase-final) and tenseness (tense vs. lax).

	ClosDur	PlatDur	ClosDisp	ClosVel
(Intercept)	47.4 (3.4)***	34.8 (2.4)***	5.3 (0.8)**	16.2 (2.2)***
positionphrase-final	3.4 (2.0)	6.2 (3.0)	−0.4 (0.2)	−2.2 (0.7)**
tensenesslax	2.5 (4.4)	−2.5 (1.9)	2.5 (1.0)	6.3 (1.9)
AIC	2754.3	3045.1	1455.8	2197.5
Num. obs.	373	381	393	393
Num. groups: participant	10	10	10	10
Num. groups: word	4	4	4	4
$R_{m}^{2}/R_{c}^{2}$	0.03/0.48	0.04/0.45	0.27/0.65	0.2/0.77

AIC: Akaike information criterion.

*** *p* < .001, **p < .01, *p < .05.

Closing duration and plateau duration of /t/ are affected neither by phrasal position nor by tenseness. This can also be seen in Figure 6(a) and (b) and is, for tenseness, in line with the acoustic closure duration (see Section 3.1.2), which tenseness does not affect either. Also, closing displacement toward /t/ is neither affected by phrasal position nor tenseness, matching the results shown in Figure 6(c). However, closing velocity is significantly slower in final positions (cf. Figures 6[d] and 7[h]). Thus, the boundary has only one effect, that is it slows the velocity of the closing movement. That durational measurements in turn do not expand significantly can be understood to be an effect of the farther distance of the gestures from the boundary.

## 4 Results summary

The main findings are summarized as follows. Acoustically, the lengthening effects are stronger at the boundary and decrease for segments farther away. The gradual, continuous local slowing effect of the π-gesture has been observed in all target words and independent of phonemic identity. Lax vowels in monosyllabic target words are significantly lengthened at phrase-final boundaries as opposed to phrase-medially. Lax vowels only show diminished stretchability in absolute terms. Contrary to the hypothesis, they are not stretched less than tense vowels in proportional terms. In the first syllable of disyllabic words, lax vowels do lengthen in absolute terms, but not significantly so. Again, they are not stretched less than tense vowels in proportional terms. Thus, the hypothesis of a diminished acoustic stretchability of lax vowels has to be rejected. The lack of significant lengthening in absolute terms in penultimate syllable might be attributed to the weaker effect of the π-gesture in this position (farther away from the boundary), which then, for the inherently shorter lax vowels, does not reach significance.

The articulatory results show, in the temporal domain, extensive and significant lengthening for the closing movement and the plateau duration. In the spatial domain, displacement and velocity vary inconsistently with respect to their boundary position and seem to be dependent in part on the phonemic identity of the consonant and of the antecedent vowel. The identity of the vowel co-varies with the tense-lax distinction, and thus influences the articulatory spatial parameters of the consonant movement. Therefore, as the data consists of only six minimal pairs, future studies are warranted to replicate the effects with an extended data set.

## 5 General discussion

We examined the effect of boundaries on lengthening of tense and lax vowels and their following consonants, testing the “uniform lengthening” hypothesis which opposes the previously established hypothesis that lax vowels stretch less than tense vowels. The latter hypothesis was derived from several studies that showed that lax vowels are generally less flexible regarding temporal adjustments. For example, Mooshammer and Fuchs (2002) and Hoole and Mooshammer (2002) found that lax vowels neither lengthen in stressed position nor shorten for fast speech. The current study found for monosyllabic words that this characteristic of lax vowels does not hold for phrase-final lengthening, but that lax vowels are acoustically longer, and that the closing gesture toward the following consonant is articulated more slowly. Contrary to the diminished stretchability hypothesis, we found that lax vowels lengthen to a similar proportion as tense vowels, which in turn supports the “uniform lengthening” hypothesis and is also the prediction made by the π-gesture model. Given these results, the notion of syllable cut prosodies (see Introduction) that assumes that lax vowels are always short because of the close contact between the vowel and the consonant (see Trubetzkoy, 1939) also has to be refuted in its strict sense, since, contrary to prominence effects, phrase-final position does lengthen closely cut (lax) vowels.

From this support of the ULH the following question arises: Why do phrasing effects override the constrained temporal inflexibility of lax vowels that was found for stress and speech tempo? Or put differently, in what aspects is phrasing different from speech rate and word stress? Let us first consider speech rate. Generally, speech rate affects vowels more than consonants (see Gay, 1981). For American English, Klatt (1973) attributed the incompressibility of lax vowels in fast speech to a threshold that does not permit further shortening of already short segments. Therefore, for speech rate, the temporal inflexibility of lax vowels seems to come about in order to avoid vowel elision and thereby maintain intelligibility. A further difference between the factors considered here is the direction of change. Whereas for stress and phrasing lengthening is expected, increase in speech rate leads to shortening.

Now, consider stress compared to phrasing. As is well known, the function of word stress and nuclear pitch accent is to increase the relative prominence compared to the adjacent units, whereas phrasing serves to chunk the speech flow into smaller prosodic units (cf. Beckman & Pierrehumbert, 1986; Cho, 2011; Keating, 2006; Lehiste, 1970). Prominence-related effects induce lengthening but also serve to enhance contrast, that is, making phonemes more dissimilar. Important phonetic correlates of contrast enhancement that have often been found are acoustic vowel expansion in stressed position (Mooshammer & Geng, 2008) and more extensive articulatory movements (see Beckman et al., 1992; Cho, 2006; Fowler, 1995). Applying the concept of contrast enhancement to tenseness could also involve that the quantity difference between tense and lax should be increased in prominent position. Therefore, only tense vowels should lengthen, but lax vowels should maintain their shortness as their most salient cue. In our data we found some evidence for that: in disyllabic words, only tense vowels lengthened significantly, whereas lax vowels did not. Since in the case of disyllabic words the stressed vowels are further away from the boundary, this could potentially be a prominence effect. As was mentioned in section 2.2, in our data phrase-medial target words carry either pre-nuclear or nuclear accents and phrase-final target words nuclear accents. We are not aware of any study that found evidence specifically for temporal differences between pre-nuclear and nuclear accents, but we can speculate that nuclear accents are more prominent and that is the reason for the asymmetrical lengthening effect on tense vowels only. Boundaries, on the other hand, mark the grouping of words into phrases. If the boundary is predominantly indicated through lengthening, then all segments need to lengthen. We therefore suggest that lax vowels stretch phrase-finally due to the function of the boundary, and thus lengthening applies to both tense and lax vowels in boundaries (but not in prominence, since prominence also serves to enhance contrast). Note though that phrase-final lengthening does not threaten the tense-lax contrast: As can be seen in Figure 3, lax vowels are still shorter than tense vowels in phrase-final positions, therefore the quantity contrast is maintained relative to the prosodic position.

A further aspect of our results that is also worth highlighting is the difference between the significant absolute lengthening (with the exception of lax vowels in disyllabic words) and the insignificant relative lengthening. While in itself this is not surprising, given the inherently different duration of the segments, it might be that this result is driven by a relatively stable perceptual target for lengthening and thus could be explained by Weber’s Law (Weber, 1834). While the data in our study do not allow us to draw any firm conclusion regarding their perception, this is a question that would be interesting to examine further.^2^

Next, we will discuss the scope of the lengthening effect. The results of the acoustic analysis show that in both mono- and disyllabic target words segments closer to the boundary lengthen more than segments farther away from it. This is especially clear in monosyllabic words, for which in every target word, codas (closest to the boundary) lengthened more than nuclei, and nuclei in turn lengthened more than onsets (see Figure 4[a]). In disyllabic words (see Figure 4[b]), /ə/ as the segment closest to the boundary lengthened by far the most. The second and the third segment away from the boundary lengthened much less, as expected, but do not show a consistent pattern. Even farther away from the boundary, the onset of the word, if it showed lengthening, lengthened the least. Overall then, the boundary effect was strongest at the boundary and reduced with distance from it, and it is continuous, as predicted by the π-gesture model (Byrd & Saltzman, 2003) and as has been found for other languages, for example, Hebrew, Dutch, Greek, English, Japanese (Berkovits, 1993b; Byrd et al., 2006; Cambier-Langeveld, 1997; Katsika, 2016; Seo et al., 2019; Shattuck-Hufnagel & Turk, 1998).

Note also that the effect of the boundary extended up to two syllables away from the boundary, up to and including the stressed syllable (while we have not examined effects even farther away, lengthening is not likely to extend beyond the two syllables, given how small and how inconsistent the lengthening on the word onset of the disyllabic words was). This is consistent with previous findings and Katsika’s theoretical account of this phenomena. Katsika et al. (2014) argue that in order to account for the earlier onset of the lengthening when stress occurs earlier in the word, the π-gesture shifts toward the π-gesture of the stressed syllable (the *μ*-gesture models stress, cf. Saltzman et al., 2008). This shift is understood to arise due to the coupling between the π-gesture and the µ-gesture. While our study has not been designed to investigate the scope of lengthening, it is worth pointing out that the interaction between boundary and stress might be language-specific, for example, stronger in German, where, based on our findings, lengthening starts robustly as early as the stressed syllable, and less strong in Greek, where, as Katsika et al. (2014) show, lengthening starts earlier when the stressed syllable occurs earlier in the word compared to when it starts later in the word but is not as robust as it is in our data.

We should also point out that the observed lengthening effects could possibly arise due to a potential difference between the pitch accent on the target word. As mentioned in Section 2.2, the target word in the phrase-medial condition could have either nuclear or pre-nuclear accent, while the target word in the boundary condition has nuclear pitch accent. However, based on the overall pattern of lengthening, namely strongest closest to the boundary and decreasing with distance from it, we think the effect is due to the boundary. Prominence effects would be expected to have a different pattern, namely strongest on the syllable nucleus (Bombien et al., 2013).

An import insight following from this study is the articulatory implementation of final lengthening. We found evidence for systematic effects of the boundary on articulatory measurements depending on vowel height and the phonemic identity of the following consonant. For three of the four investigated word pairs the change in kinematic parameters is consistent with the predictions by the mass-spring model (Beckman et al., 1992) with lengthening being produced by a spring with a smaller stiffness, evidenced by no change in displacement and lower peak velocities at phrasal boundaries. Only the word pair *Stiel/still* does not follow this pattern. The π-gesture model could account for either pattern depending on whether the reduced gestural overlap affects target achievement or not (see Byrd & Saltzman, 2003). This mixed result highlights the necessity to include a variety of phonemes in experimental studies as compared to the single vowel /ᴧ/ in Beckman et al. (1992) and to investigate articulatory data.

Our results differ from those reported in Mücke and Hermes (2007) who found almost no final lengthening in closing movements following lax vowels. In the study at hand tenseness does affect neither temporal nor spatial dimensions of the articulatory gesture toward the last consonant in the target words. There is also no evidence for a more displaced consonant closing movement in disyllabic words. One reason for these discrepancies could be that different varieties of German were considered: Viennese German in their study and Northern German in our study. Furthermore, this difference might also be attributed to methodological and individual differences since Mücke and Hermes (2007) analyzed only two speakers and the movement from the vowels /a**ː**, a/.

The next point we want to address in this discussion concerns the measurements and the relationship between the acoustic and the articulatory domain. In this study, we investigated acoustic durations as well as several kinematic parameters. Articulatory vowel duration could not be measured directly in our data because the vocalic gestures overlap and also blend with the consonantal gestures (see Browman & Goldstein, 1990; Fowler, 1980; Öhman, 1966). Therefore, we used the closing movement as the closest approximation. Connected to the kinematic analysis is an interesting observation that was not the focus of this study: As can be seen in Figure 5(b), the plateau duration of /n/ in phrase-final position was extremely long and variable, also compared to the other sonorants. It was already noticed during the articulatory labeling (cf. Section 2.3.2) that the phrase-final alveolar consonant /n/ was often not released. Thus, the tongue tip remained at the alveolar ridge during the following pause until the onset of the next utterance. The question is whether the extreme duration of the closure can be interpreted as a very pronounced final-lengthening effect or whether it may be related to other physiological events such as nasal exhalation or pre-phonatory posture. Rasskazova et al. (2018) examined the articulatory behavior during inter-speech pauses with the same data set as in this study. For cases in which the tongue tip constriction was unreleased, it was frequently found that the tongue body and the tongue back move upward toward the palate and remain there until the next segment. This dorsal movement indicates that the ongoing alveolar closure movement is not necessarily related to phrase-final lengthening, but could also be part of some kind of preparatory or pre-phonatory posture (see Gick et al., 2004; Ramanarayanan et al., 2013; Rasskazova et al., 2018).

To conclude, the results of our study do not support the predictions of the diminished stretchability hypothesis. Instead, they provide evidence for the “uniform lengthening hypothesis.” In addition, we find evidence that the effect of lengthening decreases with distance from the boundary, supporting the predictions of the π-gesture model (Byrd & Saltzman, 2003). Finally, the results from our kinematic analyses show in most cases that final lengthening is accompanied by lower peak velocities and no increase in spatial magnitude.

## Appendix 1

**Table A1.** Stimulus sentences used in this experiment.

Target	Condition	Stimulus
Bahn	phrase-medial	*Ich fuhr mit der* ***Bahn*** *am Donnerstag. Am Mittwoch wurde noch gestreikt.*
		I took the train on Thursday. On Wednesday, there was still a strike.
	phrase-final	*Ich fuhr mit der* ***Bahn***. *Am Donnerstag musste ich in Frankfurt sein.*
		I took the train. On Thursday, I had to be in Frankfurt.
Bann	phrase-medial	*Der König verhängte einen* ***Bann*** *am Donnerstag. Am Mittwoch war er vorbei.*
		The King declared the ban on Thursday. On Wednesday, it was lifted.
	phrase-final	*Der König verhängte einen* ***Bann***. *Am Donnerstag fing er an.*
		The King declared the ban. On Thursday, it started.
Beet	phrase-medial	*Sie bepflanzte ihr* ***Beet*** *am Nachmittag. Danach mähte sie den Rasen.*
		She planted her patch in the afternoon. Afterwards she mowed the lawn.
	phrase-final	*Sie bepflanzte ihr* ***Beet***. *Am Nachmittag mähte ihr Bruder den Rasen.*
		She planted her patch. In the afternoon, her brother mowed the lawn.
Bett	phrase-medial	*Er kaufte ein neues* ***Bett*** *am Nachmittag. Danach baute er es auf.*
		He bought a new bed in the afternoon. Afterwards he assembled it.
	phrase-final	*Er kaufte ein neues* ***Bett***. *Am Nachmittag baute er es auf.*
		He bought a new bed. In the afternoon, he assembled it.
Stiel	phrase-medial	*Sie kaufte Blumen mit dickem* ***Stiel*** *am Abend. Da sind sie oft im Angebot.*
		She bought the flowers with the thick stems in the evening. At that time, they are often on sale.
	phrase-final	*Sie kaufte Blumen mit dickem* ***Stiel***. *Am Abend waren sie im Angebot.*
		She bought the flowers with the thick stems. In the evening, they were on sale.
still	phrase-medial	*Das Kind war ganz* ***still*** *am Abend. Es war sehr müde.*
		The child was very quiet in the evening. It was very tired.
	phrase-final	*Das Kind war ganz* ***still***. *Am Abend war es sehr müde.*
		The child was very quiet. In the evening, it was very tired.
Ruhm	phrase-medial	*Die TV Show brachte ihr viel* ***Ruhm*** *auf der Party. Sie wurde dort auch gezeigt.*
		The TV show brought her a lot of fame at the party. It was also shown there.
	phrase-final	*Die TV Show brachte ihr viel* ***Ruhm***. *Auf der Party wurde sie auch gezeigt.*
		The TV show brought her a lot of fame. At the party it was also shown.
Rum	phrase-medial	*Sie tranken sehr viel* ***Rum*** *auf der Party. Es gab auch Whiskey.*
		They drank a lot of rum at the party. There was also some whiskey.
	phrase-final	*Sie tranken sehr viel* ***Rum***. *Auf der Party gab es auch Whiskey.*
		They drank a lot of rum. At the party there was also some whiskey.
Hüte	phrase-medial	*Sie hat viele* ***Hüte*** *bei ihrer Mutter. Da verstauben sie.*
		She has a lot of hats at her mother’s place. There they get dusty.
	phrase-final	*Sie hat viele* ***Hüte***. *Bei ihrer Mutter verstauben sie nur.*
		She has a lot of hats. At her mother’s place they just get dusty.
Hütte	phrase-medial	*Sie gingen zu der* ***Hütte*** *beim See. Danach machten sie eine Pause.*
		They went to the cabin by the lake. Afterwards they took a break.
	phrase-final	*Sie gingen zu der* ***Hütte***. *Beim See machten sie eine Pause.*
		They went to the cabin. By the lake they took a break.
Miete	phrase-medial	*Der Alex zahlt die teuerste* ***Miete*** *der Gruppe. Das gefällt ihm nicht.*
		Alex pays the most expensive rent of the group. He doesn’t like that.
	phrase-final	*Der Alex zahlt die teuerste* ***Miete***. *Der Gruppe gefällt das gut.*
		Alex pays the most expensive rent. The group likes that a lot.
Mitte	phrase-medial	*Der Alex steht in der* ***Mitte*** *der Gruppe. Das gefällt ihm nicht.*
		Alex stands in the middle of the group. He doesn’t like it.
	phrase-final	*Der Alex steht in der* ***Mitte***. *Der Gruppe gefällt das gut.*
		Alex stands in the middle. The group likes that a lot.

The target words are printed in bold.

**Table A2.** Significant contrasts for monosyllabic target words with duration as dependent variable, the predictors position (phrase-medial/-final), syllable position (O = Onset, *N* = Nucleus, C = Coda), and tenseness (tense/lax), as well as estimated value β , standard deviation (s), degrees of freedom (df), 95 % confidence intervals, and t-values, Tukey-adjusted for multiple comparisons.

	Comparison	β	s	*df*	Lower CI	Upper CI	*t*	*p*
**1**	**phrase-medial, N,tense—phrase-final, N,tense**	**−44.7**	**4.6**	**25.9**	**−61.31**	**−28.10**	**−9.64**	<.001
2	phrase-medial, N,tense—phrase-medial, C,tense	35.7	3.3	2291.0	25.00	46.38	10.92	<.001
3	phrase-medial, N,tense—phrase-final, C,tense	−28.0	4.6	25.9	−44.63	−11.42	−6.04	<.001
4	phrase-medial, N, tense—phrase-medial, N, lax	37.4	8.1	7.6	1.54	73.24	4.63	<.05
5	phrase-medial, N,tense—phrase-final, C,lax	−44.5	9.7	8.1	−86.77	−2.14	−4.57	< 0.05
6	phrase-final, N,tense—phrase-medial, C,tense	80.4	4.6	25.9	63.79	97.00	17.34	<.001
7	phrase-final, N,tense—phrase-final, C,tense	16.7	3.2	2291.0	6.15	27.21	5.18	< 0.001
8	phrase-final, N,tense—phrase-medial, N,lax	82.1	10.0	8.9	39.74	124.45	8.23	<.001
9	phrase-final, N,tense—phrase-final, N,lax	56.2	10.7	6.8	7.03	105.32	5.24	<.05
10	phrase-final, N,tense—phrase-medial, C,lax	73.1	10.0	8.9	30.75	115.46	7.33	<.01
11	phrase-medial, C,tense—phrase-final, C,tense	−63.7	4.6	25.9	−80.32	−47.11	−13.74	<.001
12	phrase-medial, C,tense—phrase-final, C,lax	−80.1	9.7	8.1	−122.47	−37.83	−8.24	<.001
13	phrase-final, C,tense—phrase-medial, N,lax	65.4	10.0	8.9	23.06	107.77	6.56	<.01
14	phrase-final, C,tense—phrase-medial, C,lax	56.4	10.0	8.9	14.07	98.78	5.66	<.01
**15**	**phrase-medial, N,lax—phrase-final, N,lax**	**−25.9**	**4.6**	**25.6**	**−42.50**	**−9.34**	**−5.60**	<.001
16	phrase-medial, N,lax—phrase-final, C,lax	−81.8	4.6	25.6	−98.42	−65.27	−17.70	<.001
17	phrase-final, N,lax—phrase-medial, C,lax	16.9	4.6	25.7	0.34	33.51	3.66	<.05
18	phrase-final, N,lax—phrase-final, C,lax	−55.9	3.2	2291.0	−66.43	−45.43	−17.42	<.001
19	phrase-medial, C,lax—phrase-final, C,lax	−72.9	4.6	25.7	−89.44	−56.27	−15.74	<.001

CI: confidence interval. The lengthening contrasts of tense and lax vowels are printed in bold.

**Table A3.** Significant contrasts for disyllabic target words with duration as dependent variable, the predictors position (phrase-medial/-final), syllable position (O1/O2 = first/second onset, N1/N2 = first/second nucleus, C = coda), and tenseness (tense/lax), as well as estimated value β, standard deviation (s), degrees of freedom (*df*), 95 % confidence intervals, and *t*-values, Tukey-adjusted for multiple comparisons.

	Comparison	β	s	*df*	Lower CI	Upper CI	*t*	*p*
1	**phrase-medial, N1,tense—phrase-final, N1,tense**	**−19.2**	**2.9**	**6.0**	**−34.02**	**−4.32**	**−6.60**	<.05
2	phrase-medial, N1,tense—phrase-medial, N2,tense	30.3	2.4	6.2	18.36	42.17	12.88	<.001
3	phrase-medial, N1,tense—phrase-final, N2,tense	−74.4	2.7	2.9	−95.43	−53.38	−27.51	<.01
4	phrase-final, N1,tense—phrase-medial, N2,tense	49.4	3.3	2.5	20.01	78.86	14.82	<.05
5	phrase-final, N1,tense—phrase-final, N2,tense	−55.2	2.4	6.4	−67.13	−43.34	−23.24	<.001
6	phrase-medial, O2,tense—phrase-final, O2,tense	−16.5	2.9	6.0	−31.36	−1.66	−5.69	<.05
7	phrase-medial, O2,tense—phrase-final, O2,lax	−21.4	3.0	5.6	−37.12	−5.70	−7.20	<.05
8	phrase-medial, O2,tense—phrase-final, N2,lax	−68.8	7.1	2.3	−135.86	−1.71	−9.70	<.05
9	phrase-final, O2,tense—phrase-medial, N2,tense	52.2	4.6	2.2	6.55	97.90	11.29	<.05
10	phrase-final, O2,tense—phrase-medial, N1,lax	51.1	5.5	2.6	3.55	98.55	9.23	<.05
11	phrase-final, O2,tense—phrase-medial, O2,lax	15.5	2.8	6.2	1.44	29.51	5.59	<.05
12	phrase-final, O2,tense—phrase-medial, N2,lax	44.7	5.1	2.7	2.60	86.80	8.76	<.05
**13**	**phrase-medial, N2,tense—phrase-final, N2,tense**	**−104.7**	**2.9**	**6.4**	**−119.44**	**−89.90**	**−35.50**	<.001
14	phrase-medial, N2,tense—phrase-final, O2,lax	−57.1	4.9	2.8	−95.74	−18.50	−11.74	<.05
15	phrase-medial, N2,tense—phrase-final, N2,lax	−104.5	8.1	2.2	−184.38	−24.62	−12.96	<.05
16	phrase-final, N2,tense—phrase-medial, N1,lax	103.5	8.3	2.3	23.98	183.02	12.53	<.05
17	phrase-final, N2,tense—phrase-medial, O2,lax	67.9	6.7	2.4	4.74	131.11	10.09	<.05
18	phrase-final, N2,tense—phrase-medial, N2,lax	97.1	8.0	2.2	17.49	176.81	12.17	<.05
19	phrase-medial, N1,lax—phrase-final, O2,lax	−56.0	5.1	2.2	−106.93	−4.97	−11.07	<.05
20	phrase-medial, N1,lax—phrase-final, N2,lax	−103.3	2.7	3.1	−123.62	−83.03	−37.66	<.001
21	phrase-final, N1,lax—phrase-final, N2,lax	−94.8	2.4	7.0	−106.60	−83.09	−39.02	<.001
22	phrase-medial, O2,lax—phrase-final, O2,lax	−20.4	2.9	6.0	−35.24	−5.52	−7.02	<.05
23	phrase-final, O2,lax—phrase-medial, N2,lax	49.6	4.6	2.2	3.91	95.29	10.72	<.05
**24**	**phrase-medial, N2,lax—phrase-final, N2,lax**	**−97.0**	**3.0**	**6.7**	**−111.64**	**−82.31**	**−32.51**	<.001

CI: confidence interval. The lengthening contrasts of tense and lax vowels are printed in bold.

## Notes

1. First, the tense-lax distinction is also quite well reflected in this speech movement (Hoole & Mooshammer, 2002; Kroos et al., 1997; Mooshammer & Fuchs, 2002, and see above). Second, although the horizontal movement of tongue back is commonly used to measure vocalic gestures (Browman & Goldstein, 1990), this is only possible in specific consonantal contexts that do not affect the tongue dorsum, such as laryngeal and bilabial contexts. Articulatory extraction of vocalic gestures in other consonantal contexts is therefore impossible due to blending and overlapping with consonant gestures. Third, using the horizontal dimension of the tongue dorsum is only an approximation since vowel positions are distinguished also by height (vertical position of dorsum and jaw) and lip rounding (Browman & Goldstein, 1990). Since our target words also consist of dorsal and alveolar consonantal contexts, we restricted our analysis to the closing gesture of the target consonant to investigate the effect of phrasal position.

2. We want to thank an anonymous reviewer and the editor for addressing these questions and pointing out a possible explanation.

## References

[bibr1-00238309211064857] Akaike H. (1974). A new look at the statistical model identification. IEEE Transactions on Automatic Control, AC-19(6), 716–723. 10.1109/TAC.1974.1100705

[bibr2-00238309211064857] Baayen R. H. (2008). Analyzing linguistic data: A practical introduction to statistics using R. Cambridge University Press.

[bibr3-00238309211064857] Barton K. (2018). MuMIn: Multi-model inference. https://CRAN.R-project.org/package=MuMIn

[bibr4-00238309211064857] Bates D. Mächler M. Bolker B. Walker S. (2015). Fitting linear mixed-effects models using lme4. Journal of Statistical Software, 67(1), 1–48. 10.18637/jss.v067.i01

[bibr5-00238309211064857] Beckman M. E. (1996). The parsing of prosody. Language and Cognitive Processes, 11(1–2), 17–68. 10.1080/016909696387213

[bibr6-00238309211064857] Beckman M. E. Edwards J. Fletcher J. (1992). Prosodic structure and tempo in a sonority model of articulatory dynamics. In Beckman M. E. Kingston J. (Eds.), Papers in Laboratory Phonology II (pp. 68–86). Cambridge University Press.

[bibr7-00238309211064857] Beckman M. E. Pierrehumbert J. B. (1986). Intonational structure in Japanese and English. Phonology, 3, 255. 10.1017/S095267570000066X

[bibr8-00238309211064857] Belz M. Rasskazova O. Krivokapić J. Mooshammer C. (2021). Interaction between phrasal structure and vowel tenseness in German [Data set]. Zenodo. 10.5281/ZENODO.4452719 PMC9975821 35021902

[bibr9-00238309211064857] Berkovits R. (1993a). Progressive utterance-final lengthening in syllables with final fricatives. Language and Speech, 36(1), 89–98. 10.1177/002383099303600105 8345773

[bibr10-00238309211064857] Berkovits R. (1993b). Utterance-final lengthening and the duration of final-stop closures. Journal of Phonetics, 21(4), 479–489.

[bibr11-00238309211064857] Berkovits R. (1994). Durational effects in final lengthening, gapping, and contrastive stress. Language and Speech, 37(3), 237–250. 10.1177/002383099403700302 7861912

[bibr12-00238309211064857] Boersma P. Weenink D. (2019). Praat: Doing phonetics by computer [Computer program version 6.1]. http://www.praat.org/

[bibr13-00238309211064857] Bombien L. Mooshammer C. Hoole P. (2013). Articulatory coordination in word-initial clusters of German. Journal of Phonetics, 41(6), 546–561. 10.1159/000442590

[bibr14-00238309211064857] Bombien L. Mooshammer C. Hoole P. Kühnert B. (2010). Prosodic and segmental effects on EPG contact patterns of word-initial German clusters. Journal of Phonetics, 38(3), 388–403. 10.1016/j.wocn.2010.03.003

[bibr15-00238309211064857] Browman C. P. Goldstein L. (1989). Articulatory gestures as phonological units. Phonology, 6(2), 201–251. 10.1017/S0952675700001019

[bibr16-00238309211064857] Browman C. P. Goldstein L. (1990). Tiers in articulatory phonology, with some implications for casual speech. In Kingston J. Beckman M. E. (Eds.), Papers in laboratory phonology I (pp. 341–376). Cambridge University Press.

[bibr17-00238309211064857] Browman C. P. Goldstein L. (1992). Articulatory phonology: An overview. Phonetica, 49(3–4), 155–180. 10.1159/000261913 1488456

[bibr18-00238309211064857] Byrd D. (2000). Articulatory vowel lengthening and coordination at phrasal junctures. Phonetica, 57, 3–16. 10.1159/000028456 10867568

[bibr19-00238309211064857] Byrd D. Krivokapić J. (2021). Cracking prosody in articulatory phonology. Annual Review of Linguistics, 7, 31–53. 10.1146/annurev-linguistics-030920-050033

[bibr20-00238309211064857] Byrd D. Krivokapić J. Lee S. (2006). How far, how long: On the temporal scope of prosodic boundary effects. The Journal of the Acoustical Society of America, 120(3), 1589–1599. 10.1121/1.2217135 17004481 PMC2423210

[bibr21-00238309211064857] Byrd D. Kaun A. Narayanan S. Saltzman E. (2000). Phrasal signatures in articulation. In Broe M. B. Pierrehumbert J. B. (Eds.), Papers in laboratory phonology V (pp. 70–87). Cambridge University Press.

[bibr22-00238309211064857] Byrd D. Riggs D. (2008). Locality interactions with prominence in determining the scope of phrasal lengthening. Journal of the International Phonetic Association, 38(2), 187–202. 10.1017/S0025100308003460 19888443 PMC2771387

[bibr23-00238309211064857] Byrd D. Saltzman E. (1998). Intragestural dynamics of multiple prosodic boundaries. Journal of Phonetics, 26(2), 173–199. 10.1006/jpho.1998.0071

[bibr24-00238309211064857] Byrd D. Saltzman E. (2003). The elastic phrase: Modeling the dynamics of boundary-adjacent lengthening. Journal of Phonetics, 31, 149–180. 10.1016/S00954470(02)00085-2

[bibr25-00238309211064857] Cambier-Langeveld T. (1997). The domain of final lengthening in the production of Dutch. Linguistics in the Netherlands, 14(1), 13–24. 10.1075/avt.14.04cam

[bibr26-00238309211064857] Cho T. (2006). Manifestation of prosodic structure in articulation: Evidence from lip kinematics in English. In Goldstein L. Whalen D. H. Best C. T. (Eds.), Laboratory phonology 8 (Vol. 8, pp. 519–548). Mouton de Gruyter.

[bibr27-00238309211064857] Cho T. (2011). Laboratory phonology. In Kula N. C. Botma B. Nasukawa K. (Eds.), The Bloomsbury companion to phonology (pp. 343–368). Continuum.

[bibr28-00238309211064857] Cho T. (2015). Language effects on timing at the segmental and suprasegmental levels. In Redford M. A. (Ed.), The handbook of speech production (pp. 505–529). Wiley. 10.1002/9781118584156.ch22

[bibr29-00238309211064857] Cho T. Keating P. A. (2001). Articulatory and acoustic studies on domain-initial strengthening in Korean. Journal of Phonetics, 29(2), 155–190. 10.1006/jpho.2001.0131

[bibr30-00238309211064857] Cho T. Kim J. Kim S. (2013). Preboundary lengthening and preaccentual shortening across syllables in a trisyllabic word in English. The Journal of the Acoustical Society of America, 133(5), EL384–EL390. 23656098 10.1121/1.4800179

[bibr31-00238309211064857] Cho T. McQueen J. M. (2005). Prosodic influences on consonant production in Dutch: Effects of prosodic boundaries, phrasal accent and lexical stress. Journal of Phonetics, 33(2), 121–157. 10.1016/j.wocn.2005.01.001

[bibr32-00238309211064857] Cooper W. E. Danly M. (1981). Segmental and temporal aspects of utterance-final lengthening. Phonetica, 38(1–3), 106–115. 10.1159/000260017

[bibr33-00238309211064857] Edwards J. Beckman M. E. Fletcher J. (1991). The articulatory kinematics of final lengthening. The Journal of the Acoustical Society of America, 89(1), 369–382. 10.1121/1.400674 2002175

[bibr34-00238309211064857] Kentner G. Féry C. (2013). A new approach to prosodic grouping. Linguistic Review, 30(2), 277–311. 10.1515/tlr-2013-0009

[bibr35-00238309211064857] Fletcher J. (2010). The prosody of speech: Timing and rhythm. In Hardcastle W. J. Laver J. Gibbon F. E. (Eds.), The handbook of phonetic sciences (pp. 524–602). Blackwell. 10.1002/9781444317251.ch15

[bibr36-00238309211064857] Fougeron C. (2001). Articulatory properties of initial segments in several prosodic constituents in French. Journal of Phonetics, 29(2), 109–135. 10.1006/jpho.2000.0114

[bibr37-00238309211064857] Fougeron C. Keating P. A. (1997). Articulatory strengthening at edges of prosodic domains. The Journal of the Acoustical Society of America, 101(6), 3728–3740. 10.1121/1.418332 9193060

[bibr38-00238309211064857] Fowler C. A. (1980). Coarticulation and theories of extrinsic timing. Journal of Phonetics, 8(1), 113–133. 10.1016/S0095-4470(19)31446-9

[bibr39-00238309211064857] Fowler C. A. (1995). Acoustic and kinematic correlates of contrastive stress accent in spoken English. In Bell-Berti F. Lawrence R. J. (Eds.), Producing speech: Contemporary issues (pp. 355–373). AIP Publishing Melville.

[bibr40-00238309211064857] Frota S. (2012). Prosodic structure, constituents and their implementation. In Cohn A. C. Fougeron C. Huffman M. (Eds.), The Oxford handbook of laboratory phonology (pp. 255–265). Oxford University Press. 10.1093/oxfordhb/9780199575039.013.0011

[bibr41-00238309211064857] Gay T. (1981). Mechanisms in the control of speech rate. Phonetica, 38(1–3), 148–158. 10.1159/000260020 7267717

[bibr42-00238309211064857] Gick B. Wilson I. Koch K. Cook C. (2004). Language-specific articulatory settings: Evidence from inter-utterance rest position. Phonetica, 61(4), 220–233. 10.1159/000084159 15824488

[bibr43-00238309211064857] Goldstein L. Whalen D. H. Best C. T. (Eds.) (2006). Laboratory phonology 8. Mouton de Gruyter. 10.1515/9783110197211

[bibr44-00238309211064857] Grice M. Baumann S. Benzmüller R. (2005). German intonation in autosegmental-metrical phonology. In Jun S.-A. (Ed.), Prosodic typology (pp. 55–83). Oxford University Press.

[bibr45-00238309211064857] Heike G. (1972). Quantitative und qualitative Differenzen von /a (:)/-Realisationen im Deutschen [Quantitative and qualitative differences of /a( : )/-realizations in German]. In Rigault A. Charbonneau R. (Eds.), Proceedings of the seventh International Congress of Phonetic Sciences / Actes du Septième Congrès international des sciences phonétiques (pp. 725–729). De Gruyter Mouton. 10.1515/9783110814750-090

[bibr46-00238309211064857] Hoole P. Mooshammer C. (2002). Articulatory analysis of the German vowel system. In Auer P. Gilles P. Spiekermann H. (Eds.), Silbenschnitt und Tonakzente (pp. 129–152). M. Niemeyer.

[bibr47-00238309211064857] Hoole P. Mooshammer C. Tillmann H. G. (1994). Kinematic analysis of vowel production in German. In Proc. 3rd International Conference on Spoken Language Processing (ICSLP 94) (pp. 53–56).

[bibr48-00238309211064857] Jørgensen H. P. (1969). Die gespannten und ungespannten Vokale in der norddeutschen Hochsprache mit einer spezifischen Untersuchung der Struktur ihrer Formantenfrequenzen [The stressed and unstressed vowels in Northern German standard speech, with a specific study of the structure of their formant frequencies]. Phonetica, 19(4), 217–245. 10.1159/000258629

[bibr49-00238309211064857] Katsika A. (2016). The role of prominence in determining the scope of boundary-related lengthening in Greek. Journal of Phonetics, 55, 149–181. 10.1016/j.wocn.2015.12.003 27773955 PMC5072286

[bibr50-00238309211064857] Katsika A. Krivokapić J. Mooshammer C. Tiede M. Goldstein L. (2014). The coordination of boundary tones and its interaction with prominence. Journal of Phonetics, 44, 62–82. 25300341 10.1016/j.wocn.2014.03.003 PMC4185973

[bibr51-00238309211064857] Keating P. A. (2006). Phonetic encoding of prosodic structure. In Harrington J. Tabain M. (Eds.), Speech production: Models, phonetic processes, and techniques (pp. 167–186). Psychology Press.

[bibr52-00238309211064857] Keating P. A. Cho T. Fougeron C. Hsu C.-S. (2004). Domain-initial articulatory strengthening in four languages. In Local J. Ogden R. Temple R. (Eds.), Phonetic interpretation (pp. 145–163). Cambridge University Press.

[bibr53-00238309211064857] Keating P. A. Shattuck-Hufnagel S. (2002). A prosodic view of word form encoding for speech production. In UCLA working papers in phonetics (pp. 112–156). UCLA Linguistics Department.

[bibr54-00238309211064857] Keating P. A. Wright R. Zhang J. (1999). Word-level asymmetries in consonant articulation. In UCLA working papers in phonetics (pp. 157–173). UCLA Linguistics Department.

[bibr55-00238309211064857] Kim J. (2020). Individual differences in the production and perception of prosodic boundaries in American English. [Unpublished doctoral dissertation, University of Michigan].

[bibr56-00238309211064857] Kim J. J. Baek Y. Cho T. Kim S. (2019). Preboundary lengthening and boundary-related spatial expansion in Korean. In Calhoun S. Escudero P. Tabain M. Warren P. (Eds.), Proceedings of the 19th International Congress of Phonetic Sciences, Melbourne, Australia (pp. 1019–1023). Australasian Speech Science and Technology Association Inc.

[bibr57-00238309211064857] Kim S. Jang J. Cho T. (2017). Articulatory characteristics of preboundary lengthening in interaction with prominence on tri-syllabic words in American English. The Journal of the Acoustical Society of America, 142(4), EL362–EL368. 10.1121/1.5005132 29092557

[bibr58-00238309211064857] Kisler T. Reichel U. D. Schiel F. (2017). Multilingual processing of speech via web services. Computer Speech & Language, 45, 326–347.

[bibr59-00238309211064857] Klatt D. H. (1973). Interaction between two factors that influence vowel duration. The Journal of the Acoustical Society of America, 54(4), 1102–1104. 10.1121/1.1914322 4757455

[bibr60-00238309211064857] Kohler K. J. (1983). Prosodic boundary signals in German. Phonetica, 40, 89–134. 10.1159/000261685

[bibr61-00238309211064857] Krivokapić J. (2007). Prosodic planning: Effects of phrasal length and complexity on pause duration. Journal of Phonetics, 35(2), 162–179. 10.1016/j.wocn.2006.04.001 18379639 PMC2131696

[bibr62-00238309211064857] Krivokapić J. (2014). Gestural coordination at prosodic boundaries and its role for prosodic structure and speech planning processes. Philosophical Transactions of the Royal Society. Series B, Biological Sciences, 369(1658). 10.1098/rstb.2013.0397 PMC4240964 25385775

[bibr63-00238309211064857] Kroos C. Hoole P. Kühnert B. Tillmann H. G. (1997). Phonetic evidence for the phonological status of the tense-lax distinction in German. In Forschungsberichte des Instituts für Phonetik und Sprachliche Kommunikation (FIPKM) (pp. 17–25).

[bibr64-00238309211064857] Kuznetsova A. Brockhoff P. B. Christensen R. H. B. (2017). lmerTest package: Tests in linear mixed effects models. Journal of Statistical Software, 82(13), 1–26. 10.18637/jss.v082.i13

[bibr65-00238309211064857] Lehiste I. (1970). Suprasegmentals. MIT Press.

[bibr66-00238309211064857] Lehiste I. (1973). Phonetic disambiguation of syntactic ambiguity. The Journal of the Acoustical Society of America, 53(1), 380. 10.1121/1.1982702 4765807

[bibr67-00238309211064857] Lenth R. (2019). emmeans: Estimated Marginal Means, aka Least-Squares Means. https://CRAN.R-project.org/package=emmeans

[bibr68-00238309211064857] Michalke M. (2017). sylly.de: Language Support for “sylly” Package: German. https://github.com/unDocUMeantIt/sylly

[bibr69-00238309211064857] Mooshammer C. Fuchs S. (2002). Stress distinction in German: Simulating kinematic parameters of tongue-tip gestures. Journal of Phonetics, 30(3), 337–355. 10.1006/jpho.2001.0159

[bibr70-00238309211064857] Mooshammer C. Geng C. (2008). Acoustic and articulatory manifestations of vowel reduction in German. Journal of the International Phonetic Association, 38(2), 117–136. 10.1017/S0025100308003435

[bibr71-00238309211064857] Mücke D. Hermes A. (2007). Phrase boundaries and peak alignment: An acoustic and articulatory study. In Proceedings of ICPhS XVI, Saarbrücken, Germany (pp. 997–1000).

[bibr72-00238309211064857] Nakagawa S. Schielzeth H. (2013). A general and simple method for obtaining R2 from generalized linear mixed-effects models. Methods in Ecology and Evolution, 4(2), 133–142. 10.1111/j.2041-210x.2012.00261.x

[bibr73-00238309211064857] Nakai S. Turk A. E. Suomi K. Granlund S. Ylitalo R. Kunnari S. (2012). Quantity constraints on the temporal implementation of phrasal prosody in Northern Finnish. Journal of Phonetics, 40(6), 796–807. 10.1016/j.wocn.2012.08.003

[bibr74-00238309211064857] Öhman S. E. G. (1966). Coarticulation in VCV utterances: Spectrographic measurements. The Journal of the Acoustical Society of America, 39(1), 151–168. 10.1121/1.1909864 5904529

[bibr75-00238309211064857] Oller D. K. (1973). The effect of position in utterance on speech segment duration in English. The Journal of the Acoustical Society of America, 54(5), 1235–1247. 10.1121/1.1914393 4765808

[bibr76-00238309211064857] Peters B. (2003). Multiple cues for phonetic phrase boundaries in German spontaneous speech. In 15th International Congress of Phonetic Sciences, Barcelona, Spain, August 3–9 [ICPhS-15] (pp. 1795–1798).

[bibr77-00238309211064857] Petrone C. Truckenbrodt H. Wellmann C. Holzgrefe-Lang J. Wartenburger I. Höhle B. (2017). Prosodic boundary cues in German: Evidence from the production and perception of bracketed lists. Journal of Phonetics, 61, 71–92. 10.1016/j.wocn.2017.01.002

[bibr78-00238309211064857] R Core Team. (2018). R: A language and environment for statistical computing. R Foundation for Statistical Computing.

[bibr79-00238309211064857] Ramanarayanan V. Goldstein L. Byrd D. Narayanan S. S. (2013). An investigation of articulatory setting using real-time magnetic resonance imaging. The Journal of the Acoustical Society of America, 134(1), 510–519. 10.1121/1.4807639 23862826 PMC3724797

[bibr80-00238309211064857] Rasskazova O. Mooshammer C. Fuchs S. (2018). Articulatory settings during inter-speech pauses. In Belz M. Mooshammer C. Fuchs S. Jannedy S. Rasskazova O. Żygis M. (Eds.), Proceedings of the Conference on Phonetics & Phonology in German-speaking countries (P&P 13), Berlin (pp. 161–164). 10.18452/18805

[bibr81-00238309211064857] Rusaw E. C. (2013). Modeling temporal coordination in speech production using an artificial central pattern generator neural network [PhD thesis, University of Illinois at Urbana-Champaign, Champaign, IL].

[bibr82-00238309211064857] Saltzman E. L. Munhall K. G. (1989). A dynamical approach to gestural patterning in speech production. Ecological Psychology, 1(4), 333–382.

[bibr83-00238309211064857] Saltzman E. L. Nam H. Krivokapić J. Goldstein L. (2008). A task-dynamic toolkit for modeling the effects of prosodic structure on articulation. In Proceedings of the 4th international conference on speech prosody (speech prosody 2008), Campinas, Brazil (pp. 175–184).

[bibr84-00238309211064857] Savariaux C. Badin P. Samson A. Gerber S. (2017). A comparative study of the precision of Carstens and Northern Digital Instruments Electromagnetic Articulographs. Journal of Speech, Language, and Hearing Research, 60(2), 322–340. 10.1044/2016JSLHR-S-15-0223 28152131

[bibr85-00238309211064857] Sendlmeier W. F. (1981). Der Einfluss von Qualität und Quantität auf die Perzeption betonter Vokale des Deutschen [The influence of quality and quantity on the perception of stressed vowels in German]. Phonetica, 38(5–6), 291–308. 10.1159/000260034 7330094

[bibr86-00238309211064857] Seo J. Kim S. Kubozono H. Cho T. (2019). Preboundary lengthening in Japanese: To what extent do lexical pitch accent and moraic structure matter? The Journal of the Acoustical Society of America, 146(3), 1817–1823. 31590553 10.1121/1.5122191

[bibr87-00238309211064857] Shattuck-Hufnagel S. Turk A. E. (1996). A Prosody tutorial for investigators of auditory sentence processing. Journal of Psycholinguistic Research, 25(2), 193–247. 10.1007/BF01708572 8667297

[bibr88-00238309211064857] Shattuck-Hufnagel S. Turk A. E. (1998). The domain of phrase-final lengthening in English. In Kuhl P. K. (Ed.), The sound of the future: A global view of acoustics in the 21st century, 16th International Congress on Acoustics and 135th meeting, Woodbury, Acoustical Society of America (pp. 1235–1236).

[bibr89-00238309211064857] Sievers E. (1901). Grundzüge der Phonetik zur Einführung in das Studium der Lautlehre der indogermanischen Sprachen [Fundamentals of phonetics for the introduction to the study of phonetics of Indo-European languages]. (5th ed., Vol. 1). Breitkopf & Härtel. https://archive.org/details/grundzgederphon00sievgoog/page/n9

[bibr90-00238309211064857] Smiljanic R. Bradlow A. R. (2008). speaking styles in English and Croatian. Stability of temporal contrasts across. Journal of Phonetics, 36(1), 91–113. 10.1016/j.wocn.2007.02.002 19122747 PMC2390829

[bibr91-00238309211064857] Tabain M. (2003). Effects of prosodic boundary on/aC/ sequences: Articulatory results. The Journal of the Acoustical Society of America, 113(5), 2834–2849. 10.1121/1.1523390 12765400

[bibr92-00238309211064857] Tabain M. Perrier P. (2005). Articulation and acoustics of /i/ in pre-boundary position in French. Journal of Phonetics, 33(1), 77–100. 10.1016/j.wocn.2004.04.003

[bibr93-00238309211064857] Tiede M. (2005). MVIEW: Software for visualization and analysis of concurrently recorded movement data. Haskins Laboratories.

[bibr94-00238309211064857] Trubetzkoy N. S. (1938). Die phonologischen Grundzüge der sogenannten “Quantität” in den verschiedenen Sprachen [The basic phonological features of the so-called “quantity” in different languages]. In Hoepli U. (Ed.), Scritti in onore di Alfredo Trombetti (pp. 155–174). Milano.

[bibr95-00238309211064857] Trubetzkoy N. S. (1939). Grundzüge der Phonologie [Fundamentals of phonology] (Vol. 7). Prag.

[bibr96-00238309211064857] Turk A. E. Shattuck-Hufnagel S. (2000). Word-boundary-related duration patterns in English. Journal of Phonetics, 28(4), 397–440. 10.1006/jpho.2000.0123

[bibr97-00238309211064857] Turk A. E. Shattuck-Hufnagel S. (2007). Multiple targets of phrase-final lengthening in American English words. Journal of Phonetics, 35(4), 445–472. 10.1016/j.wocn.2006.12.001

[bibr98-00238309211064857] Turk A. E. White L. (1999). Structural influences on accentual lengthening in English. Journal of Phonetics, 27(2), 171–206. 10.1006/jpho.1999.0093

[bibr99-00238309211064857] Vaissière J. (1983). Language-independent prosodic features. In Cutler A. Ladd D. R. (Eds.), Prosody: Models and measurements (pp. 53–66). Springer.

[bibr100-00238309211064857] Vennemann T. (1991). Skizze der deutschen Wortprosodie [A sketch of the German word prosody]. Zeitschrift für Sprachwissenschaft, 10(1), 86–111. 10.1515/zfsw.1991.10.1.86

[bibr101-00238309211064857] Wagner M. Watson D. G. (2010). Experimental and theoretical advances in prosody: A review. Language and Cognitive Processes, 25(7–9), 905–945. 10.1080/2F01690961003589492 22096264 PMC3216045

[bibr102-00238309211064857] Weber E. H. (1834). De pulsu, resorptione, auditu et tactu. Annotationes anatomicae et physiologicae [On pulse, resorption, sense of hearing and sense of touch. Anatomical and physiological notes]. Leipzig, C. F. Koehler.

[bibr103-00238309211064857] Weirich M. Simpson A. P. (2015). Impact and interaction of accent realization and speaker sex on vowel length in German. In Leemann A. Kolly M.-J. Schmid S. Dellwo V. (Eds.), Trends in phonetics and phonology (pp. 109–123). Peter Lang.

[bibr104-00238309211064857] White L. (2002). English speech timing: A domain and locus approach [Unpublished doctoral dissertation, University of Edinburgh].

[bibr105-00238309211064857] Wightman C. W. Shattuck-Hufnagel S. Ostendorf M. Price P. J. (1992). Segmental durations in the vicinity of prosodic phrase boundaries. The Journal of the Acoustical Society of America, 91(3), 1707–1717. 10.1121/1.402450 1564206

[bibr106-00238309211064857] Winkelmann R. Harrington J. Jänsch K. (2017). EMU-SDMS: Advanced speech database management and analysis in R. Computer Speech & Language, 45, 392–410. 10.1016/j.csl.2017.01.002

[bibr107-00238309211064857] Winkelmann R. Jaensch K. Cassidy S. Harrington J. (2016). emuR: Main Package of the EMU Speech Database Management System. https://rdrr.io/cran/emuR/

